# Ethnobotanical study on edible flowers in Xishuangbanna, China

**DOI:** 10.1186/s13002-023-00608-1

**Published:** 2023-09-30

**Authors:** Qing Zhang, Zhuo Cheng, Yanxiao Fan, Dezheng Zhang, Miaomiao Wang, Jihai Zhang, Sarana Sommano, Xianjin Wu, Chunlin Long

**Affiliations:** 1 Key Laboratory of Ecology and Environment in Minority Areas (Minzu University of China), National Ethnic Affairs Commission, Beijing, 100081 China; 2 College of Life and Environmental Sciences, Minzu University of China, Beijing, 100081 China; 3 School of Ethnology and Sociology, Yunnan University, Kunming, 650091 China; 4 Key Laboratory of Ethnomedicine (Minzu University of China), Ministry of Education, Beijing, 100081 China; 5 Faculty of Agriculture, Chiang Mai University, Chiang Mai, 50200 Thailand; 6 Key Laboratory of Research and Utilization of Ethnomedicinal Plant Resources of Hunan Province, College of Biological and Food Engineering, Huaihua University, Huaihua, 418000 China; 7 Institute of National Security Studies, Minzu University of China, Beijing, 100081 China

**Keywords:** Biocultural diversity conservation, Edible flowers, Ethnobotany, Food and medicine continuum, Traditional knowledge

## Abstract

**Background:**

Edible flowers (EFs) represent valuable sources of both food and medicinal resources, holding the promise to enhance human well-being. Unfortunately, their significance is often overlooked. Ethnobotanical studies on the EFs are lacking in comparison with their botanical and phytochemical research. The practice of consuming flowers as food has a rich culture and long history in China, especially among different linguistic groups in Xishuangbanna, Yunnan. However, economic activities have led to a decline of this tradition. Consequently, preserving the traditional knowledge and culture tied to the EFs in Xishuangbanna becomes both essential and pressing.

**Methods:**

The field ethnobotanical survey was conducted in Xishuangbanna during five visits in April 2021 and May 2023, covering 48 villages and 19 local markets of all three county-level areas and 9 different linguistic groups. By conducting a comprehensive literature review and on-site field surveys, relevant information regarding the EFs of Xishuangbanna was systematically collected and documented. Additionally, the relative frequency of citation (RFC) values were calculated from the survey data.

**Results:**

A total of 212 taxa (including species and varieties) of EFs from 58 families and 141 genera were documented in the study area. The edible parts of flowers were classified into 13 categories including peduncle, petal, flower buds, inflorescence as a whole, and etc. They were consumed in 21 ways and as 8 types of food. The inflorescence was the most commonly consumed category, accounting for 85 species (40.1%) of the total categories. They always eat flowers as vegetables (184 species, 86.8%). The preparing form of stir-frying was the preferred food preparation method (138, 65.1%). The Xishuangbanna locals had profound knowledge of which EFs required specific processing to remove their toxicity or bitterness. The dishes can be made from either exclusively from the flowers themselves or by incorporating them alongside other plant parts like stems and leaves. Some EFs with high RFC value, such as *Musa acuminata* and *Bauhinia variegata* var. *candida*, showed significant cultural meanings. These edible flowers occupy specific positions in local traditional culture.

**Conclusion:**

Traditional knowledge regarding edible flowers holds substantial significance and serves as a representative element of the flower-eating culture in Xishuangbanna. Nevertheless, this knowledge and cultural practice are currently decreasing. Serving as a bridge between tradition and modernity, the flower-eating culture, which derives from local people’s practical experience, shows the potential of EFs and can be applied to the conservation of biocultural diversity, healthy food systems, and sustainable development.

## Introduction

Throughout the long history of survival and development, mankind has developed a close relationship with plants [1]. Approximately 13% genera of vascular plants are available as dietary food, followed by ornamental plants (26%) and medicines (16%) [2]. As the archetype of biocultural diversity, food plants establish a connection between the environment and biodiversity, while also bridging human society with cultural richness [3]. Numerous edible flowers are integral to the gastronomic heritage of various countries and play a crucial role in human nutrition and food security [4–7].

Edible flowers (EFs) are broadly defined as plants in which entire flower organs or their components are deemed edible. These flowers serve various purposes, such as in medicine, flavor extraction, and as essential ingredients within the food and medicinal sectors [8, 9]. It is reported that there are 180 species of common EFs, belonging to 97 families and 100 genera, globally consumed in all kinds of food and drinks [10]. Research and review articles on its nutritional and phytochemical elements have always increased [11]. Studies show that many EFs are rich of protein, vitamins, minerals, fiber and carbohydrates. A total of 302 bioactive compounds including flavonoids, terpenoids, phenylpropanoids, alkaloids and organic acids, with 22 biological activities were summarized [12–14]. Nowadays, EFs have transformed into valuable sources of both sustenance and medicinal benefits, capable of enhancing people’s well-being. Regrettably, their traditional importance frequently goes unnoticed or is overlooked [13–15]. Some ethnobotanical studies on EFs have demonstrated that their cultural attributes hold the potential to contribute to local economies, the preservation of biodiversity, and the advancement of rural development [16–19]. Nonetheless, the quantity of such studies is notably lower in comparison with the extent of their phytochemical research.

Flowers have been utilized by Chinese people for a long time in various ways, including their consumption as a source of sustenance. Some 2500 years ago, the Chinese poet Qu Yuan wrote about eating flowers in his poem ‘Li Sao’: “朝饮木兰之坠露兮, 夕餐秋菊之落英”, which means taking dew drops falling from magnolia flowers as a drink in the morning, and taking petals falling from chrysanthemums as a meal in the evening. This is the earliest record of Chinese flower-eating culture [20].

In Yunnan Province, which is rich in both biodiversity and cultural diversity, there are more than 300 EF species consumed by different linguistic people there. Those species belonged to 74 families and 178 genera [21]. Depending on their habits, living environment and altitude, the species of flowers chosen for consumption vary greatly from one group to another. The diversity of edible parts of flowers consumed, and the processing and cooking methods reflected rich EFs’ traditional knowledge [21, 22].

Xishuangbanna is home to a wide variety of edible plants, many of which have been consumed for centuries by local communities for food and medicinal purposes [23, 24]. It is also an important ethnobotany’s birthplace in China. The first ethnobotanical paper in China published in 1982 focused on useful plants in Xishuangbanna, where 7 EFs were recorded. These included *Bauhinia variegata* L., *Sesbania grandiflora* (L.) Pers., *Buddleja officinalis* Maxim., *Gmelina arborea* Roxb., *Elsholtzia rugulosa* Hemsl., *Smilax zeylanica* L. and *Musa* spp. [25]. Previous ethnobotanical investigations revealed that the floral components of food plants are prominently utilized as edibles in the region, with a minimum of 23 EFs being commonly employed by the local population [24, 26]. The culture of EFs is a crucial part of traditional knowledge and practices of different linguistic groups in Xishuangbanna such as the Dai, Hani, Jinuo, Yao, Lahu, Lisu, Yi, and Han communities residing in that area [27]. The long history of flower-eating and the rich variety of EF is the result of local people’s adaptation to their ecological environment [28].

In conjunction with the previously mentioned list of the EFs, an extensive ethnobotanical literature search encompassing edible plants in Yunnan and beneficial flora in Xishuangbanna was conducted through reputable online scientific databases including Google Scholar, Sci Finder, Web of Science, Springer Link, PubMed, Wiley, as well as Chinese databases like CNKI, Baidu Academic, and Chinese Science and Technology Periodical databases. The keywords such as “edible flowers,” “eating flowers,” “traditional use of flowers,” “flowers culture,” “edible plants,” and “food plants” were used. The publications were filtered for the Chinese, English and Japanese. Based on the literature review, we found that there are many edible flower species distributed in Xishuangbanna. The local people have the tradition to collect and consume EFs. Unfortunately, it lacks documentation of flower food and associated traditional knowledge, or systematic ethnobotanical investigation.

Eating flowers was once a common practice in human society. However, with the development of society, especially the popularity of fast food among the young generation, the flower-eating culture has gradually disappeared in many countries [29]. The flower-eating culture prevalent among Yunnan societies reflects the local people’s deep understanding of plants and nature, containing folk ecological wisdom and key clues to the sustainable development of local communities [30]. In recent decades, rapid economic development happened in China. In particular, massive tourism and commercial plantations of rubber and tropical fruits have emerged in Xishuangbanna. These economic activities have resulted in the rapid decline of the flower-eating culture. It is necessary and urgent to preserve traditional knowledge associated with EFs in Xishuangbanna. The edible flower in this article refers to the floral parts utilized by people for food, drink, or food supplementary material purposes. It may be flower only, or flower with other parts of the plant when consumed. The main purposes of this study were to (1) document the EFs consumed by different linguistic groups in Xishuangbanna, (2) reveal the flower-eating culture there, and (3) propose strategies for its conservation and future development.

## Methods

### Study area

**Fig. 1:**
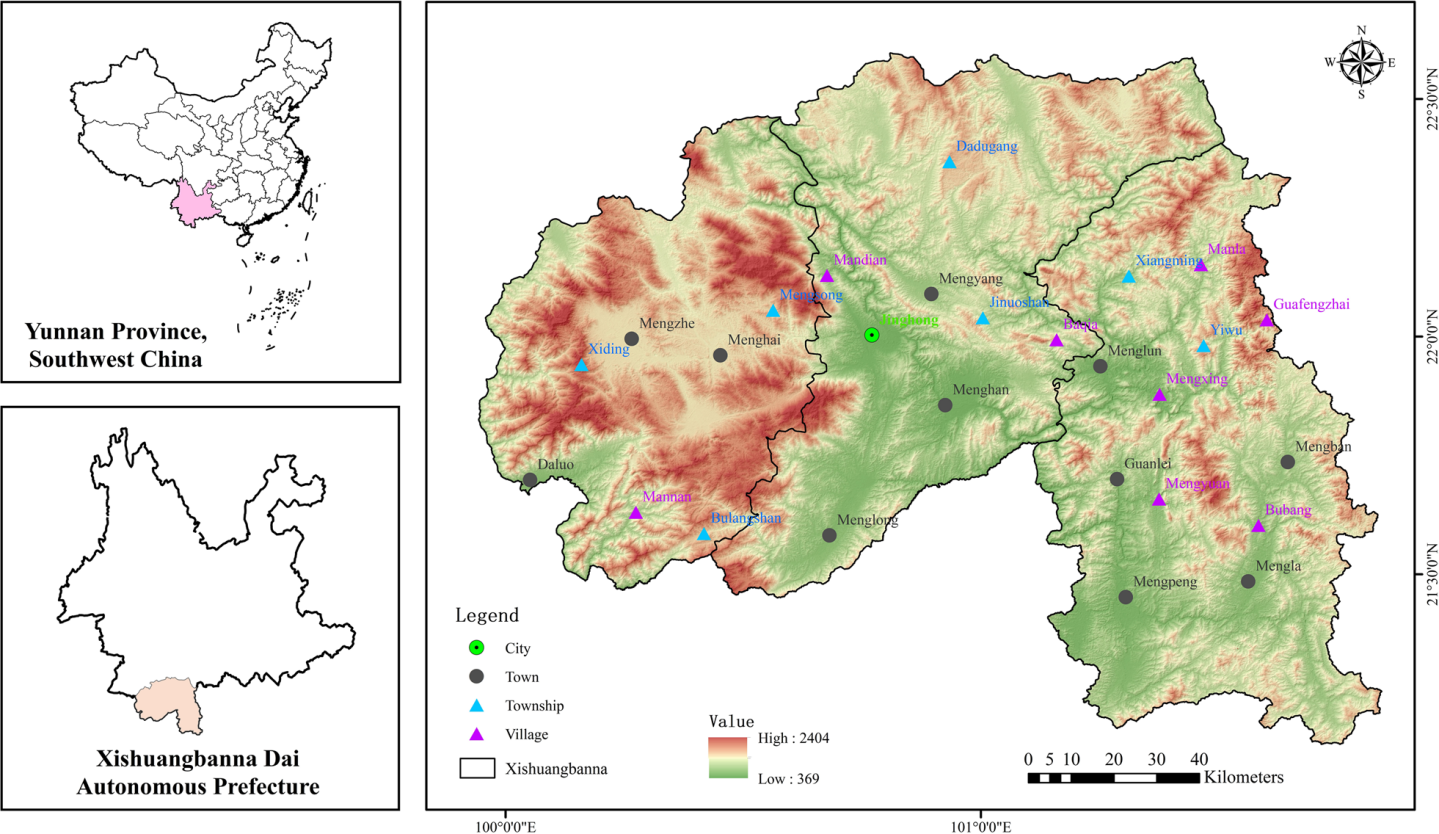
The study area. The colored map is Xishuangbanna Prefecture, in which markets in towns/townships and villages investigated are marked with black dots and blue/pink triangles

Xishuangbanna Dai Autonomous Prefecture is the study area of the present paper. It is located in the southernmost part of Yunnan Province, China. The Lancang-Mekong River flows through this prefecture from the north to the south, and the whole territory belongs to its watershed. The total land area of the prefecture is more than 19,000 km^2^, bordering Laos and Myanmar to the south (Fig. 1). It has tropical monsoon climate with an average annual temperature of 15.1–21.7 °C. The annual precipitation varies from 1000 to 2500 mm [31]. Throughout the year, the region maintains a warm and humid climate, characterized by the absence of harsh cold during winter, the absence of extreme heat in summer, and favorable light conditions. Xishuangbanna is the home to the largest tropical forest in China, with an area of approximately 15,500 km^2^ (81% forest coverage) [28]. The geographical environment and rainforest climatic conditions have nurtured rich local plant resources, with nearly 5000 species of higher plants, accounting for about 16% of China’s higher plant species, making it one of the areas with the richest biodiversity in China [32].

Xishuangbanna Prefecture, under the jurisdiction of Jinghong City, Mengla County and Menghai County, has been a multi-ethnic settlement since ancient times. In addition to the Dai people, there are 12 other linguistic groups, including the Han, Hani, Lahu, Bulang, Yi, Jinuo, Yao, Wa and Hui. The ethnic minority population is about 790,000, or 69.97% of the total population, of which the Dai are the most numerous, accounting for about one-third of the total population [33]. In the ancient Dai language, “Xishuangbanna” means “ideal and magical land of happiness” [28]. Each ethnic group has its own language and traditional culture as well as its way of perceiving and using local natural resources, creating a rich cultural diversity in Xishuangbanna [24].

The Dai people live in the basins and lowlands of Xishuangbanna, with a population of 334,500. They have their own oral and writing language belonging to the Zhuang-Dai branch, the Zhuang-Dong language group, Sino-Tibetan family. The Dai people believe the Hinayana Buddhism and animism. They grow sticky rice in the paddy fields and manage homegardens. The glutinous rice is their staple food. Vegetables are mostly grown in homegardens, while wild food plants are collected as supplements (www.xsbn.gov.cn, accessed 21 August 2023).

The Hani people live in the mountains of Xishuangbanna. They have other names such as Aini and Akha, with a population of 211,800. The Hani do not have written language. Their spoken language belongs to the Yi branch, Tibetan-Myanmar language group, Sino-Tibetan family. They grow rice, upland crops such as maize and tea. It is common for the Hani people to collect wild food plants. They believe animism and worship ancestors (www.xsbn.gov.cn, accessed 21 August 2023).

There are 62,100 Lahu people living in Xishuangbanna. They speak Lahu language, which belongs to the Yi branch, Tibetan-Myanmar language group, Sino-Tibetan language family. Most Lahu people believe animism, but some of them believe Buddhism. They live in the mountains and practice upland farming. Collecting edible plants become one of their essential activities.

The Yi people live in the Northeastern part of Xishuangbanna, with a population of 59,500 (www.xsbn.gov.cn, accessed 21 August 2023). They have oral and written language, belonging to the Yi branch, Tibetan-Myanmar language group, Sino-Tibetan family. The Yi people believe animism and worship ancestors. They grow upland crops and collect edible plants in the mountains.

There are 52,800 Bulang people living in Xishuangbanna (www.xsbn.gov.cn, accessed 21 August 2023). They speak their own language but do not have written characters. Their language belongs to the Bulang branch, Mon Khmer group, South Asian language family. They live in the uplands of Menghai County, believe the Hinayana Buddhism. It is essential for the Bulang people to manage teagardens and collect wild edible plants to support their lives.

Most Jinuo people concentrate to live in Jinuo Township of Jinghong City, with a population of 25,800 (www.xsbn.gov.cn, accessed 21 August 2023). They grow upland crops while collecting becomes important livelihood form. The Jinuo people believe animism, and worship their ancestors.

The Yao people live in the mountainous areas of eastern Xishuangbanna, with a population of 23,700 (www.xsbn.gov.cn, accessed 21 August 2023). They do not have written language. Their spoken language belongs to Yao branch, Miao-Yao group, Sino-Tibetan language family. The Yao people grow upland crops and collect wild edible plants for food.

In addition to the Han Chinese, a few thousands of Miao, Wa, Zhuang, Jingpo and Hui people also live in Xishuangbanna. The Hui ran good business, while the others earn their livelihoods based on agricultural production. Most Han people in the prefecture migrated from Hunan Province in 1960s to clear lands for rubber plantations.

Traditionally, the Hani, Bulang, Wa, Yao, Miao, Yi, Lahu and Jinuo people who lived in the mountainous areas practiced shifting cultivation (slash-and-burn agriculture). They cultivated upland rice, taro (*Colocasia esculenta*), buckwheat and beans, but rarely grew vegetables. The ancestors of uplanders and Han Chinese in Xishuangbanna had planted tea seedlings in natural forests. The old tea gardens scattered in the prefecture can still produce high-quality tea products called Pu-er tea.

Nowadays, all people in Xishuangbanna have gotten rid of poverty and lived at a higher-level livelihoods. The locals who live in lowlands earn incomes from rubber and tropical fruit productions, while those in uplands from tea production. Also, the tourism in Xishuangbanna brings a lot of opportunities to the local people. In general, their economic status is at the middle level in the country. However, the locals still collect a lot of wild edible plants from the forests, farming lands, roadsides and wetlands for daily lives. It becomes a custom or traditional culture to gather food plants from the wild lands.

### Field survey and data collection

The field ethnobotanical surveys were conducted in Xishuangbanna during five visits in 2021 and May 2023. It covered 48 villages and 19 markets (Fig. 1) in all three county-level areas, Menghai and Mengla, and Jinghong in the prefecture. Different linguistic groups including Dai, Hani, Bulang, Jinuo, Yao, Miao, Lahu, Yi, Wa, Lisu and Han Chinese were involved.

The maps were downloaded from the official site (http://bzdt.ch.mnr.gov.cn/). The investigation sites including markets and villages were marked based on ArcGIS 10.7.

In villages, free listing, key informant interviews and participatory observation were conducted [34, 35]. In total, 201 people ranging from 16 to 87 years old were interviewed during the field surveys, including 92 females. The informants were mostly interviewed in local markets, reaching 128 people consisting of 65 females and 63 males (Table 1). Among these respondents, 24 people were selected as the key informants. In the context of this article, EFs are defined as floral components consumed by individuals for culinary, beverage, or supplementary dietary purposes. Consumption may involve the flower alone or in combination with other plant parts. The interviews were conducted using a semi-structured approach, covering topics such as EF species, their pronunciation, edible parts, processing techniques, medicinal advantages of consuming the flowers, additional uses of the consumed plant, and the motivations behind their consumption.

**Table 1:** Demographic characteristics of the interview respondents

Locality	Jinghong	Menghai	Mengla	Subtotal
Groups*	D/H/J/Ot	D/H/B/Ot	D/H/Y/Ot	
*Markets*				
Male	8/6/3/5	6/5/2/3	7/8/6/4	63
Female	7/3/3/7	7/3/3/4	12/4/4/8	65
*Villages*				
Male	5/3/3/4	2/3/2/4	8/6/3/3	46
Female	2/2/2/2	2/1/1/2	4/3/2/4	27
Total	65	50	86	201

*D Dai, *H* Hani, *J* Jinuo, *B* Bulang, *Y* Yao, *Ot* Others

The linguistic group, age, education level and occupation of informants were also recorded. Before the interviews, each informant who participated in was informed of the purpose of the project, and their consent was obtained.

The availability of the EFs influenced by seasons and processing, making comprehensive coverage challenging in limited field surveys [19]. A pivotal reference for this study was the comprehensive investigation by Prof. Yitao Liu and Prof. Chunlin Long (one of the corresponding authors of this paper), pioneers in the field of edible flowers in Yunnan [21, 22]. Local market was the entrance of local food system with the largest concentration of flower-eating plants in the region [3]. They were also gathering places for traditional knowledge from the stallholders and consumers [17]. In our survey, we examined 19 markets within an urban area and 11 towns across Xishuangbanna. Our interviews encompassed EFs' vendors as well as select consumers purchasing these items. The traded flower-eating plants in the markets and supplementary insights shared by local individuals (both vendors and consumers) were meticulously documented.

The flowers collected and consumed by the local people in Xishuangbanna can be divided into different categories according to their edibility of different parts of the flower. A single flower consists of bract, pedicel, receptacle, calyx, petal and corolla, stamens (filament, anther and pollen), pistils (ovary, style and stigma), nectars, and sometimes appendix. In many cases, there are inflorescence consisting of flowers. People consume flowers together with leaves and stems if all these parts are edible or taste better. But sometimes only a single part of flower (for example, petal or pedicel), or male flower only, is selected for food. Therefore, the edible parts of EFs were recorded and calculated in the market surveys and field investigations.

The multiple uses of flowers including their cultural values were also investigated. Some species of EFs consumed as food can be used for different purposes such as ornamentals, medicine and pigment. The EFs have contributed to the development of local culture in Xishuangbanna. Their values with cultural significance were also investigated and recorded, in addition to their role as food.

The voucher specimens were collected through the process of conducting market surveys or en route to subsequent locations. The nomenclature of all vascular plants follows Plants of the World Online (Kew) (powo.science.kew.org, accessed on 16–17 August 2023) and World Flora Online (www.worldfloraonline.org, accessed on 23–28 May 2023). Prof. Chunlin Long and Zhuo Cheng identified the plant species, and the voucher specimens were deposited in the herbarium at Minzu University of China, Beijing.

### Quantitative analysis

Quantitative analysis of the data was conducted to understand the diversity of edible flower species in Xishuangbanna. It will help to evaluate the potential of traditional knowledge of the target communities. Thus, the number of species, the number of respondents who provide information, and information on the edible flower species were presented based on the citations. Our quantitative analysis was followed by ethnobotanical indexes using relative frequency of citation.

The relative frequency of citation (RFC) in this study was used to evaluate the local importance of EFs in Xishuangbanna. The formula for RFC is *RFC = FC/N*, which means the number of respondents mentioning an EF divided by the total number of respondents [36]. *FC *is the number of respondents who gave citations at each species, and* N* is the number of respondents. The index varies from 0 to 1. A higher RFC value means more local people know the EF [37]. The RFC values for each EF are added to the database.

## Results

### Species diversity of EFs in Xishuangbanna

Based on our field surveys and literature review, 212 taxa (including species and varieties) belonging to 58 families and 141 genera are documented as being used as EFs in Xishuangbanna. Their vernacular names, Chinese names, distributions in Yunnan, habitats, used parts, use types, methods of preparation for food, additional uses, RFC values and voucher numbers are also listed in Table 2.

**Table 2:** Inventory of edible flowers consumed by different linguistic groups in Xishuangbanna, Yunnan, China

Scientific name	Family	Vernacular name (D: Dai language; H: Hani; JN: Jinuo; L: Lahu; W: Wa; Y: Yi; YN: Yunnan dialect)	Chinese name	Distribution in Yunnan	Habitat	Flower parts used for food	With/without other parts when collecting/ consuming flowers	Categories of use type	Methods of preparation for food	Additional use(s)	RFC	Voucher number
*Aglaia odorata* Lour	Meliaceae	ge mai yon yo (D)	米仔兰	S Yunnan	Cultivated	Flower, flower buds	Flower only	Tea-scenting flower	Scenting tea	Flowers used for promoting qi circulation and relieving depression, sobering up alcohol and clearing the lungs, and for extracting essential oils	0.008	BN412
*Alcea rosea* L.	Malvaceae	Yi zhang hong (YN)	蜀葵	S Yunnan	Cultivated	Petal	Flower only	Vegetable	Cooked in water with chicken or pork; congee-making	Whole plant for ornamental. Roots, stems, leaves, flowers and seeds as medicine for clearing heat, harmonizing the blood and moistening the intestines	0.008	BN058
*Allium chinense* G. Don	Amaryllidaceae	huo huang (D)	藠头	Whole Yunnan	Cultivated	Inflorescence	With leaves	Vegetable	Stir-fried; pickled	Common vegetable and also for medicinal purpose. Invigorating stomach	0.004	BN021
*Allium fistulosum* L.	Amaryllidaceae	pa bang (D), huo pa ge huo lai (H)	葱	Whole Yunnan	Cultivated	Inflorescence	With leaves	Vegetable, seasoning	Stir-fried; salad; pickled	Common vegetable, seasoning and also for medicinal purpose. Inducing resuscitation	0.008	BN033
*Allium hookeri* Thwaites	Amaryllidaceae	luo wo mo (Y), pi cai (YN)	宽叶韭	Whole Yunnan	Wild	Inflorescence	Flower only	Vegetable	Stir-fried with pork or eggs; cooked in water; pickled	Common vegetable. For dispelling blood stasis and promoting blood circulation, promoting qi circulation and promoting stagnation	0.048	BN066
*Allium sativum* L.	Amaryllidaceae	huo huang (D), dun bie (Y)	蒜	Whole Yunnan	Cultivated	Inflorescence	Flower only	Vegetable, seasoning	Stir-fried; pickled	Common vegetable, seasoning and also for medicinal purpose. Bulbs for anti-inflammation and rheumatism	0.056	BN010
*Allium tuberosum* Rottler ex Sprengle	Amaryllidaceae	pa biang (D), xiu we (L), dong bu lai (Y)	韭	Whole Yunnan	Cultivated	Inflorescence	Flower only	Vegetable	Stir-fried; soup; salad; pickled	Common vegetable, seasoning and also for medicinal purpose. Diminishing inflammation, relieving cough, expelling phlegm and nourishing liver and kidney	0.048	BN003
*Alpinia blepharocalyx* K. Schum	Zingiberaceae	ja bi ne mi bo we (L), guc ga (D), me du/niu ma me du (H), me le me du (JN)	云南草蔻	S & W Yunnan	Wild	Inflorescence	Flower only	Vegetable, seasoning	Stir-fried; roasted; salad; as seasonings	Fruits for food	0.008	BN213
*Alpinia emaculata* S. Q. Tong	Zingiberaceae	ou ga mi bo we (L)	无斑山姜	S Yunnan	Wild	Inflorescence	Flower only	Seasoning	As seasonings	Rhizome for invigorating stomach	0.004	BN075
*Alpinia galanga* (L.) Willd	Zingiberaceae	mi bo wei (L), me pi (H), guo ha/guo ha pie/guo he kou you (D)	红豆蔻	S Yunnan	Wild	Inflorescence	Flower only	Vegetable, seasoning	As seasonings; salad	Roots, stems and fruits for medicine	0.008	BN311
*Alpinia oxyphylla* Miq	Zingiberaceae	ja bi ne mi bo we (L)	益智	S Yunnan	Cultivated	Inflorescence	Flower only	Seasoning	As seasonings	Seeds boiled in water and consumed as medicine	0.008	BN237
*Alpinia platychilus* K. Schumann	Zingiberaceae	bi ga/guo ga long (D)	宽唇山姜	S Yunnan	Wild	Flower buds	Flower only	Vegetable	Stir-fried; soup; roasted; cooked with bamboo shoots or other vegetables	Seeds for stomach distention, indigestion, nausea and vomiting, diarrhea, generalized soreness caused by cold wind. Fruits for stomachache	0.004	BN198
*Alpinia roxburghii* Sweet	Zingiberaceae	ja bi ne mi bo we (L), xiao cao kou (YN)	绿苞山姜	S Yunnan	Wild	Flower buds	Flower only	Vegetable, seasoning	Cooked in water; salad; as seasonings	Seeds for warm stomach	0.004	BN101
*Alpinia zerumbet* (Pers.) B. L. Burtt & R. M. Sm	Zingiberaceae	La yue ge (JN)	艳山姜	Tropical Yunnan	Wild	Flower	Flower only	Vegetable	Stir-fried; salad	Used to treat indigestion, vomiting, and diarrhea	0.024	BN248
*Amaranthus cruentus* L.	Amaranthaceae	na wo we (L)	老鸦谷	Whole Yunnan	Wild, cultivated	Inflorescence	Always with young shoots and tender leaves	Vegetable	Stir-fried; cooked in water; salad	Wild leafy vegetable. Seeds are edible after brewing. Clearing heat-toxin	0.008	BN043
*Amaranthus spinosus* L.	Amaranthaceae	wo chu na mi (L), pa la (D), me du (JN)	刺苋	S Yunnan	Wild	Inflorescence, flower buds	Always with young shoots and tender leaves	Vegetable	Stir-fried; cooked in water; salad	Wild leafy vegetable. Stems and leaves for medicine	0.008	BN074
*Amaranthus tricolor* L.	Amaranthaceae	na wo we (L)	苋菜	Whole Yunnan	Cultivated	Inflorescence	Always with young shoots and tender leaves	Vegetable	Stir-fried; cooked in water; salad	Common leafy vegetable. Improving eyesight	0.016	BN034
*Amaranthus viridis* L.	Amaranthaceae	na m (L), pa hon ge (D), wo ji (H), ya chi ge ye (JN)	皱果苋	S Yunnan	Wild	Inflorescence	Always with young shoots and tender leaves	Vegetable, seasoning	Stir-fried; cooked in water; salad	Wild leafy vegetable. Whole plant for medicine to clear heat-toxin and promote diuresis. Decoction of roots and stems for hypertension	0.016	BN157
*Amomum compactum* Solander ex Maton	Zingiberaceae	yi bu ri we (L), bai doukou (YN)	爪哇白豆蔻	S Yunnan	Cultivated	Flower buds	With young shoots	Seasoning	Salad; as seasonings	Roots, stems and fruits for medicine	0.004	BN258
*Amomum koenigii* J. F. Gmelin	Zingiberaceae	lei wu se, chu bo we (L)	野草果	S Yunnan	Wild	Inflorescence, flower buds	With young shoots	Seasoning	As seasonings or making sauce	Roots, stems and fruits for medicine	0.004	BN226
*Amomum maximum* Roxb	Zingiberaceae	guo gu/ma gu (D), me ko (H), ja bi li m bo (L), mie qie (JN)	九翅豆蔻	S Yunnan	Wild, cultivated	Inflorescence	With young shoots	Vegetable, seasoning	Cooked in water; as seasonings	Rhizome or whole plant boiled in water for dyspepsia, stomach diseases and hepatitis. Fruits for food and medicine	0.008	BN137
*Amorphophallus yuloensis* H. Li	Araceae	La bong (JN)	攸乐磨芋	S Yunnan	Wild	Inflorescence	Inflorescence only	Vegetable	Stir-fried after blanching in hot water	Tuber used as food	0.008	BN041
*Amorphophallus yunnanensis* Engl	Araceae	Bu lai duo (JN)	滇磨芋	S Yunnan	Wild	Inflorescence	Inflorescence only	Vegetable	Stir-fried after blanching in hot water	Tuber used as food and medicine	0.016	BN036
*Apium graveolens* L.	Apiaceae	a xiu ka ma (L)	旱芹	Whole Yunnan	Cultivated	Inflorescence	Always with young stems and leaves	Vegetable	Stir-fried; salad after blanching; pickled	Wild leafy vegetable. Whole plant for medicine to clear heat-toxin, cool blood and lower blood pressure. Antihypertension	0.016	BN204
*Areca catechu* L.	Palmae	guo ma bu (D), mao lao si (H), Bin nan (YN)	槟榔	S Yunnan	Cultivated	Male inflorescence	Male part only	Vegetable	Stewed with pork	Fruits for chowing and medicine. Expelling phlegm and relieving cough	0.008	BN091
*Arisaema erubescens* (Wall.) Schott	Araceae	a cao ba (L)	一把伞南星	S Yunnan	Wild	Spathe and scape	Flower only	Vegetable	Pickled after drying in the sun, well-cooked for detoxifying	Tubers for treating burns by fire	0.004	BN315
*Averrhoa carambola* L.	Oxalidaceae	ma fen (D), bao mo ya bo(JN)	阳桃	Tropical Yunnan	Cultivated	Flower	Flower only	Vegetable	Stewed with duck meat	Tropical fruits. Nourishing kidney and lung	0.004	BN039
*Bauhinia variegata* var. *candida* (Roxb.) Voigt	Leguminosae	mai xiu bu/luo mei hao (D), pa wo we (L), du pia/doub a ye/du biu (H), ge le ping (W), jie bo/bo fa (JN), beng bo lun fang (Y), lao baihua (YN)	粉花羊蹄甲; 老白花	S Yunnan	Wild	Flower buds, all parts of flower, sometimes only petal, calyx	Flower only, also young leaves and pods	Vegetable	After blanching in hot water, stir-fried; cooked in water; fried; salad; stewed	Common wild vegetable in S Yunnan. Flowers, young leaves and pods consumed as vegetable. Roots used as anthelminthica and for relieving dyspepsia. Extracts can relieve hemorrhoids, constipation and lepriasis	0.086	BN018
*Begonia cucullata* Willd	Begoniaceae	Hai tang hua (YN)	四季海棠	Most areas of Yunnan	Cultivated	Flower	Sometimes with leaves	Vegetable	Stir-fried with other food; salad	Ornamental	0.016	BN202
*Benincasa hispida* (Thunb.) Cogn	Cucurbitaceae	ma ha meng (D), tuo pu lu (JN)	冬瓜	Whole Yunnan	Cultivated	Flower	Flower only	Vegetable	Stir-fried	Fruit is a common vegetable. The decoction of fruit for fever, cold, menstrual disorder and diuresis. Seeds and melon skin for medicine	0.004	BN350
*Boehmeria nivea* (L.) Gaudich	Urticaceae	ban tarng (D)	苎麻	S Yunnan	Cultivated	Inflorescence	Always with young leaves	Vegetable	Stir-fried; cooked in water; as hot-pot vegetable	The seed oil is edible and roots for medicine	0.008	BN235
*Bombax ceiba* L.	Malvaceae	re bie (JN), nie wei/la mai we (L), bo ming fang (Y), ge luo zhong (D), pan zhi hua (YN)	木棉	Tropical Yunnan	Wild, cultivated	Stamens, petal, sometimes all parts of flowers	Flower only	Vegetable	After blanching in hot water, stir-fried; cooked in water; salad after blanching; tea substitute; stewed; soup	Whole plant for medicine to clear heat-toxin, promote diuresis and hemostasis. Seed fibers for beddings	0.056	BN053
*Brassica campestris* var. *purpuraria* L.H.Bariley	Cruciferae	zi cai hua (YN)	红菜薹	C Yunnan	Imported	Inflorescence	Sometimes with stems and leaves	Vegetable	Stir-fried; cooked in water	Leafy vegetable	0.016	BN233
*Brassica juncea* (L.) Czernajew	Cruciferae	wo pa ho we (L)	冲菜	Whole Yunnan	Cultivated	Inflorescence	Always with young stems and leaves	Vegetable	Stir-fried; cooked in water; pickled	Common leafy vegetable. Seeds for suppressing hyperactive liver and hemostasis	0.048	BN026
*Brassica oleracea* L.	Cruciferae	pa ga lang (D), gai lan (YN)	芥蓝	Whole Yunnan	Cultivated	Inflorescence	Always with stems and leaves	Vegetable, tea substitute	Stir-fried; cooked in water	Many cultivators are common leafy vegetables and famous oil-bearing crops. Also used as medicine for detoxifying and expelling wind, and relieving summer heat	0.068	BN019
*Brassica oleracea* var. *botrytis* L.	Cruciferae	wo li we (L), cai hua (YN)	花椰菜	Whole Yunnan	Cultivated	Inflorescence	Flower only	Vegetable	Stir-fried; cooked in water	Common vegetable	0.086	BN011
*Brassica oleracea* var. *italica* Plenck	Cruciferae	qing hua cai (YN)	西蓝花	Whole Yunnan	Cultivated	Inflorescence	Flower only	Vegetable	Stir-fried; cooked in water	Common vegetable	0.056	BN040
*Brassica rapa* var. *chinensis* (L.) Kitamura	Cruciferae	da hou (W)	青菜	Whole Yunnan	Cultivated	Inflorescence	Always with young stems and leaves	Vegetable	Stir-fried; cooked in water	Common leafy vegetable. Also used for expectorant and tourniquet relieving, detoxifying and swelling reducing	0.056	BN028
*Brassica rapa* var. *glabra* Regel	Cruciferae		白菜	Whole Yunnan	Cultivated	Inflorescence	Always with young stems and leaves	Vegetable	Stir-fried; cooked in water	Common vegetable	0.056	BN030
*Brassica rapa* var*. oleifera* de Candolle	Cruciferae	paga langmang (D), you cai (YN)	芸薹	Whole Yunnan	Cultivated	Inflorescence	Always with stems and leaves	Vegetable	Fried; cooked in water; pickled; making cakes	Many cultivators are common leafy vegetables and famous oil-bearing crops. Also used as medicine for cooling blood, dispersing blood, detoxifying and reducing swelling	0.024	BN047
*Buddleja officinalis* Maxim	Scrophulariaceae	se ni we be be (L), huo mi huo xe/a bu bu shi le (JN), duo tuan/kao lun, mo ma gun (D), o pao pao xiu (H), lan wang fang/ nanr lang (Y), baba hua(YN)	密蒙花	Most areas of Yunnan	Wild	Inflorescence, nectar	Flower only	Tea substitute, fooddye, snack	Cooked in water; tea substitute; dying rice and other foods; collecting nectars as snack	Common edible flower. Flowers for extracting perfume oils. Clearing heat and promoting diuresis. Improving eyesight	0.056	BN123
*Calystegia hederacea* Wall	Convolvulaceae	lao mu zhu cao (YN)	打碗花	Whole Yunnan	Wild	Flower	Flower only	Vegetable	Stir-fried	Roots for irregular menstruation	0.004	BN092
*Camellia japonica* L.	Theaceae	zha fang (Y), bai yang cha (YN)	华东山茶	S & W Yunnan	Cultivated	Petal	Flower only	Vegetable, tea substitute, snack	Tea substitute; cooked in water; stir-fried; making soup or sweet	Roots and seed oils for medicine. Clearing heat-toxin, detumescence, cooling blood for hemostasis	0.004	BN224
*Camellia oleifera* Abel	Theaceae	la nan mang (D)	油茶	S Yunnan	Cultivated	Flower	Flower only	Vegetable, tea substitute	Tea substitute; cooked in water; stir-fried	Roots, seeds and seed oils for medicine. Clearing heat-toxin	0.004	BN362
*Camellia reticulata* Lindl	Theaceae	niu mo gu we (L)	滇山茶	S Yunnan	Cultivated	Flower	Flower only	Vegetable, tea substitute	Tea substitute; cooked in water; stir-fried	Clearing heat-toxin, detumescence, cooling blood for hemostasis	0.004	BN263
*Camellia sinensis* var. *assamica* (J. W. Masters) Kitamura	Theaceae	yi la (D), pe liu (L), luo bo (JN)	普洱茶	S Yunnan	Cultivated	Flower	Flower only; also young leaves	Tea substitute	Making tea or congee; cooked in water; brewing	Young leaves and stems can be cooked. Leaves for medicine. Clearing heat-toxin and cooling blood for hemostasis	0.016	BN054
*Canna indica* L.	Cannaceae	man dong (D), niu a bu we (L), ba jiao yu (YN)	美人蕉	Whole Yunnan	Cultivated	Nectar	Flower only	Snack	Salad; collecting nectar as sweetener	Many horticultural cultivars for ornamental. Canna indica ‘Edulis’ is a common forage crop and for extracting starch as well	0.016	BN130
*Capsella bursa-pastoris* (L.) Medic	Cruciferae	a ga wo ma (L)	荠菜	Whole Yunnan	Wild, occasionally cultivated	Flower	Always with leaves	Vegetable	Salad; stir-fried; cooked in water	Wild vegetable. Used as medicine for treating dysentery, gonorrhea, edema, chyluria, hematemesis, bloody stools, blood avalanche, excessive menstruation, and eye pain	0.016	BN185
*Carica papaya* L.	Caricaceae	ma bao (JN), po me we (L)	番木瓜	S Yunnan	Cultivated	Female inflorescence, male flower buds	Flower only	Vegetable	Salad with spice and sauce	Common tropical fruit. Used as medicine for improving breast milk secretion	0.008	BN063
*Cassia fistula* L.	Leguminosae	guo long liang/guo long (D)	腊肠树	S Yunnan	Cultivated	Petal	Flower only	Vegetable	Stir-fried	The young leaves, fruit pods and fruits are edible. Fruits mashed for constipation and labyrinthitis	0.004	BN351
*Celosia argentea* L.	Amaranthaceae	luo lai guo ma (D), an na zhi we (L)	青葙	C, W & S Yunnan	Cultivated	Flower, flower buds	Flower only	Vegetable	Stir-fried; cooking congee or soup; tea substitute	Young stems, leaves and seeds for food.Whole plant for eliminating dampness, clearing heat, destroying parasites and hemostasis. Roots for promoting blood circulation. Flowers for ornamental	0.008	BN183
*Celosia cristata* L.	Amaranthaceae	an ra zhi we (L), mam zham fang(Y)	鸡冠花	Whole Yunnan	Cultivated	Inflorescence	Flower only	Vegetable	Stir-fried with pork; cooked in water; stewed; soup	Cooling blood for hemostasis and nourishing blood	0.016	BN221
*Chrysanthemum morifolium* Ramat	Asteraceae	ak se e we (L)	菊花	Whole Yunnan	Cultivated	All parts of flower, sometimes petal	Flower only	Vegetable,tea substitute, snack, beverages	Salad; stir-fried; cooked in water; steamed; fried; baked; stewed; tea substitute	Clearing heat-fire, suppressing hyperactive liver and improving eyesight	0.016	BN029
*Citrus maxima* (Burm.) Merr	Rutaceae	ma nu ma we (L), le (W), pao guo (YN)	柚	S Yunnan	Cultivated	All parts of flower	Flower only	Vegetable, tea-scenting flower	Cooked in water; pickled; salad; scenting tea; as seasoning	Regulating qi-flowing, removing phlegm and relieving pain	0.008	BN190
*Citrus medica* L.	Rutaceae	ma vei (D), fo shou (YN)	香橼	S Yunnan	Cultivated	Flower only	Flower only	Vegetable, tea substitute	Stir-fried; as seasoning	Regulating qi-flowing for relieving pain.Resolving food stagnation. Removing phlegmregulating qi to relieve pain, dissipating digestion and resolving phlegm	0.008	BN146
*Clerodendrum bungei* Steud	Lamiaceae	guo bing (D), buo huo chi (H), yu gi (JN)	臭牡丹	C & S Yunnan	Wild	Inflorescence	With stems and leaves	Vegetable	After blanching, roasted in banana leaves; stir-fried; cooked in water	Expel wind and activate blood flow, clear away heat and toxic materia	0.016	BN167
*Clerodendrum fortunatum* L.	Lamiaceae	Deng long cao (YN)	白花灯笼	S Yunnan	Cultivated	Petal	Flower only	Vegetable	Stir-fried; cooked in water	Ornamental. Used as medicine to treat cold, sore throat, cough, injuries caused by falls	0.008	BN268
*Clerodendrum japonicum* (Thunb.) Sweet	Lamiaceae	bin liang (D), na pe na ce da (L), bie bo a bo(JN)	赪桐	S Yunnan	Wild	Inflorescence	With leaves	Vegetable	After blanching, roasted in leaves; stir-fried; cooked in water; steamed; salad	Whole plants for dispelling pathogenic wind, removing dampness, detumescence, eliminating blood stasis and harmonizing qi	0.008	BN071
*Clerodendrum serratum* var. *amplexifolium* Moldenke	Lamiaceae	na pe nao e da (L), guang san ka (D), ni ya (H)	三台花	Tropical Yunnan	Wild	Inflorescence	Flower only	Vegetable	Roasted; stir-fried; cooked in water; mixed with mashed potato; salad after blanching in hot water	Used as medicine to treat damp heat dysentery, gonorrhea syndrome, rheumatism	0.008	BN295
*Clitoria ternatea* L.	Leguminosae	Nanbeo (D)	蝶豆	S Yunnan	Cultivated	Flower	Flower only	Vegetable, fooddye, tea substitute	Stir-fried; cooked in water; tea substitute; fried; dying beverage	Flowers for dyeing	0.016	BN143
*Colocasia esculenta* (L.) Schott	Araceae	bei xi we (L), buo pe/wen buo (D), pun bu lai (JN), bei wu/bu ma a ye (H), hou (Y)	芋	Whole Yunnan	Cultivated	Peduncle, spathe, all parts of flower	Flower only	Vegetable	After removing the peel of petiole, stir-fried; stewed; pickled; steaming with eggplants	Common food crop. Leaves and corm are edible. Leaves can be used as fodder	0.056	BN035
*Crassocephalum crepidioides* (Benth.) S. Moore	Asteraceae	mu no we (L), pa yar mo (D), guan dong wei niu(H), miao kuo (JN), ge ming cai (YN)	野茼蒿	Whole Yunnan	Wild	Flower	With young leaves	Vegetable	Stir-fried; cooked in water; fried; after blanching as salad	Whole plants for for clearing heat for arresting cough and strengthening the stomach	0.016	BN142
*Curcuma aromatica* Salisb	Zingiberaceae	Wan jie long, wan lie (D)	郁金	S Yunnan	Wild, cultivated	Peduncle	Peduncle only	Vegetable	Stir-fried; cooked in water	Ornamental. Rhizomes for medicine	0.016	BN348
*Curcuma longa* L.	Zingiberaceae	wang huo long/hao ming/hao min gie yin (D), me xiu (H), nie she (JN)	姜黄	Tropical Yunnan	Cultivated	Inflorescence	With rhizomes	Vegetable	Roasted; cooked in water; stewed; fried	Rhizome soaked in wine as dressing for rhizomestomach diseases, injury, skin diseases,joint pain, itching, inflammation. Rhizome for activating qi to resolve stagnation and removing blood stasis to relieve pain	0.008	BN064
*Cucurbita moschata* (Duch. ex Lam.) Duch. ex Poiret	Cucurbitaceae	ma ba leng/log ba (D), pe me wei (L), bu lu shi bier (W), tuo ke le/u du ar pa ar bu (JN), ging gua fang (Y)	南瓜	Whole Yunnan	Cultivated	Peduncle, mostly male flowers, occasionally female flowers	Flower only	Vegetable	Removing the peel of peduncle, stir-fried; cooked in water; soup	Young stem, leaf, flower and fruit are edible. Seeds for snacks. Clearing heat and promoting diuresis, detumescence, eliminating blood stasis and anti-cancer	0.036	BN056
*Cynara scolymus* L.	Asteraceae	yang ji (YN)	菜蓟	Part of Yunnan	Cultivated	Receptacle	Flower only	Vegetable	After boiling in hot water, salad or stir-fried	Assisting treatment of cardiovascular diseases	0.008	BN286
*Dendrobium chrysotoxum* Lindl	Orchidaceae	nuo ma hai (D)	鼓槌石斛	S & W Yunnan		Flower	Flower only	Vegetable, tea substitute	Cooked with eggs; salad; cooked in water; stewed; tea substitute	Stems for nourishing yin, promoting fluid production, quenching thirst and moistening lung. Dispersing stagnated liver qi for relieving qi stagnation and relieving spasm, pain and fatigue	0.016	BN013
*Dendrobium dixanthum* Rchb	Orchidaceae	Diao lanhua (YN)	黄花石斛	S Yunnan		Flower	Flower only	Vegetable	Stir-fried; cooked in water	Moistening lung invigorating and benefiting Qi	0.004	BN269
*Dendrobium moniliforme* (L.) Sw	Orchidaceae	Tong pi shihu (YN)	细茎石斛	Tropical Yunnan		Flower	Flower only	Vegetable	Stir-fried; cooked in water	Moistening lung invigorating and benefiting Qi	0.008	BN1093
*Dendrobium nobile* Lindl	Orchidaceae	Da cai (JN)	石斛	S & W Yunnan		Flower	Flower only	Vegetable, tea substitute	Stir-fried; cooked in water; tea substitute; stewed	Moistening lung invigorating and benefiting Qi. Stems for antidote. Promoting stomach intestine motility. Flowers for ornamental	0.008	BN105
*Dendrobium officinale* Kimura et Migo	Orchidaceae	Zignat awlmurku caq natzhid (L)	铁皮石斛	Part of Yunnan	Cultivated	Flower	Flower only	Vegetable, tea substitute	Stir-fried with eggs; cooked in water; tea substitute	Stem used as medicine for a long history to treat many ailments, and improving immunity	0.008	BN042
*Dregea sinensis* Hemsl	Apocynaceae	Ya gai xian dai /da bai gai (D), nai jiang hua (YN)	苦绳	C Yunnan	Wild, imported	Inflorescence	Sometimes with a few young leaves	Vegetable	Stir-fried; cooked in water; fried with pork or ham	The whole plant used for dispelling wind and dampness, relieving cough and asthma, relieving inflammation and lactation, reducing swelling and pain, diuresis	0.016	BN188
*Dregea volubilis* (L. f.) Benth. ex Hook. f	Apocynaceae	a ka ba a ba/teng ke ou ni (H), ye ya/gong gai fang (Y), lao mei hong/log pa ong (D), pa ke lou ar bu (JN)	南山藤	S Yunnan	Wild, cultivated	Inflorescence	Sometimes with a few young leaves	Vegetable	Stir-fried; cooked in water; fried; salad after blanching in hot water	A common leafy vegetable. Whole plant for clearing heat, eliminating dampness and relieving pain	0.056	BN037
*Eichhornia crassipes* (Mart.) Solme	Pontederiaceae	水葫芦, pa bu dun (D), lao niu wa miu (H), yi gan ga mu go (L)	凤眼蓝	Whole Yunnan	Escaped	Inflorescence	With young leaves	Vegetable	Stir-fried; salad; salad after blanching in hot water	Whole plant as fodder for pigs or medicine. Nourishing intestine for relaxing bowels	0.016	BN369
*Elsholtzia bodinieri* Vaniot	Lamiaceae	mixikakewe (L)	东紫苏	C & S Yunnan	Wild	Inflorescence	With leaves	Tea substitute	Tea substitute	Clearing heat-toxin	0.016	BN201
*Elsholtzia ciliata* (Thunb.) Hyland	Lamiaceae	mi xi ka ke we (L), ye la you ma (D), pu dao zha (Y), lao bo mo hao (H), sao ba cha (YN)	香薷	S Yunnan	Wild	Inflorescence	With leaves	Tea substitute	Tea substitute	Clearing heat-toxin	0.024	BN135
*Elsholtzia communis* (Coll. et Hemsl.) Diels	Lamiaceae	Xiang zi su (YN)	吉龙草	Part of Yunnan	Cultivated	Inflorescence	Always with stems and leaves	Tea substitute	Tea substitute	Whole plant used to treat throat swelling and pain, breast sores, swollen gums, cold	0.004	BN326
*Epiphyllum oxypetalum* (DC.) Haw	Cactaceae	Tan hua (YN)	昙花	Whole Yunnan	Cultivated	All parts of flower, sometimes petal	Flower only	Vegetable	Stewed with eggs or pork	Clearing lung-heat for arresting cough, cooling blood for hemostasis and tranquilizing by nourishing the heart	0.008	BN293
*Eranthemum pulchellum* Andrews	Acanthaceae	可爱花, bie bu (JN)	喜花草	S & SE Yunnan		All parts of flower	Flower only	Vegetable	Stir-fried; cooked in water; soup	Roots and leaves for medicine	0.008	BN182
*Erythrina variegata* L.	Leguminosae	mai duan (D), hei suo (H)	刺桐	E, S & W Yunnan		All parts of flower	Flower only	Vegetable	Stir-fried after blanching in hot water; cooked in water	Treating wounds caused by metal instruments and hemostasis	0.008	BN173
*Etlingera elatior* (Jack) R. M. Sm	Zingiberaceae	mo liang bu/mo liang long/mo diu meng (D), mie bu mie nai (JN)	火炬姜	S Yunnan	Cultivated	Young inflorescence	Flower only	Vegetable, seasoning	Stir-fried; salad; cooked in water	Rhizome for dyspepsia, nauseanausea, stomach diseases. The flower essences are also used to promote physical and mental well-being	0.008	BN145
*Fagopyrum esculentum* Moench	Polygonaceae	Ha lei (H)	甜荞	Whole Yunnan	Cultivated	Inflorescence	Always with young leaves	Vegetable	Stir-fried; cooked in water	Roots and stems for medicine	0.008	BN150
*Fagopyrum tataricum* (L.) Gaertn	Polygonaceae	pa hon shong (D)	苦荞	Whole Yunnan	Cultivated	Inflorescence	Always with young leaves	Vegetable	Stir-fried; cooked in water	Seeds and leaves for food. Roots and stems for medicine	0.004	BN249
*Ficus auriculata* Lour	Moraceae	bu a ba(H), meng luo biu (Y), se bu (JN), ma gua (D), xi bo xi (L), xiang er duo (YN)	大果榕	SE, S & SW Yunnan	Cultivated	Inflorescence (Hypanthium)	Sometimes with young fruits	Vegetable, snack	Salad; cooked in water	Young stems, leaves and barks as well as fruits can be eaten.Fruits for promoting lactation and benefiting qi for promoting production of blood	0.024	BN102
*Ficus carica* L.	Moraceae	ne gu xi (L), si bu (H)	无花果	C & S Yunnan		Inflorescence (Hypanthium)	Sometimes with young fruits	Vegetable, snack	Salad	Roots and leaves for medicine	0.024	BN285
*Ficus oligodon* Miq	Moraceae	pa wa (D), xi gu ma shiao (H)	苹果榕	S Yunnan		Inflorescence (Hypanthium)	Sometimes with young fruits	Vegetable, snack	Salad	Young stem and leaf can be eaten	0.024	BN177
*Ficus racemosa* L.	Moraceae	ouluse (JN), xiboxi (L), pula (W), mailei (D), xibubuh (H), amo (Y)	聚果榕	S Yunnan	Cultivated	Inflorescence (Hypanthium)	Sometimes with young fruits	Vegetable, snack	Salad	Leaves can be cooked as vegetable	0.024	BN186
*Foeniculum vulgare* Mill	Apiaceae	jin ji/pa ji (D)	茴香	Whole Yunnan	Cultivated	Inflorescence	Always with leaves	Vegetable, seasoning	Stir-fried; cooked in water; as seasonings	The powder of leaves and roots for epilepsy and dyspepsia. Relieving pains, expelling phlegm, anti-inflammatory and diuresis	0.016	BN088
*Gardenia jasminoides* Ellis	Rubiaceae	Muo suai long (D), zhi zi hua (YN)	栀子	Whole Yunnan	Wild, cultivated	Petal	Flower only	Vegetable, tea substitute	Stir-fried; cooked in water; tea substitute	Ornamental. Aromatic and dye crop. Used as medicine for treating Jaundice, gonorrhea syndrome and other diseases	0.016	BN147
*Gmelina arborea* Roxb	Lamiaceae	guouo/mai suo (D), lu mei ar bu (JN); se jie/shi jie a ye (H); yang bie fang (Y)	云南石梓	S Yunnan	Wild, cultivated	All parts of flower	Flower only	Snack, fooddye	Steamed after grinding, together with sticky rice powder	Stems for housebuilding timber and cooking utensils. For gynecological stasis and rheumatic arthralgia	0.056	BN087
*Haymondia wallichii* (DC.) A. N. Egan & B. Pan bis	Leguminosae	Ge hua (YN)	须弥葛	S Yunnan	Wild	Inflorescence	Flower only	Vegetable	Pickled	Forage tree and shellac host tree	0.008	BN376
*Hedychium coccineum* Smith	Zingiberaceae	Dai heng hao, nuodai huomu (D)	红姜花	S Yunnan	Cultivated	Inflorescence	Flower only	Vegetable	Stir-fried; cooked in water	Ornamental. Used as medicine for treating cough, asthma, constipation, low back pain, limb numbness and others	0.024	BN297
*Helianthus annuus* L.	Asteraceae	mo wan wai (D)	向日葵	Whole Yunnan	Cultivated	Petal	Flower only	Vegetable	Stir-fried; cooked in water	Seeds are used to make vegetable oil. Whole plant for medicine and ornamental	0.008	BN175
*Hemerocallis fulva* var. *aurantiaca* (Baker) M. Hotta	Asphodelaceae	Jin zhen cai (YN)	黄花菜	Whole Yunnan	Cultivated or escaped	Flower	Flower only	Vegetable	Stir-fried; cooked in water; rosted; fried	Ornamental. Whole plant used for nourishing, cooling blood, calming mind and improving eyesight, brain strengthening, and anti-aging	0.016	BN059
*Heterosmilax polyandra* (Gagnep.) P. Li & C. X. Fu	Smilacaceae		多蕊肖菝葜	S Yunnan	Wild	Inflorescence	Flower only	Vegetable	Stir-fried	Fruit is edible	0.008	BN323
*Hibiscus mutabilis* L.	Malvaceae	nuo san ran (D)	芙蓉花	S Yunnan	Cultivated	All parts of flower, mostly petal	Flower only	Vegetable	Cooked in water; making congee	Clearing lung-heat	0.008	BN283
*Hibiscus rosa-sinensis* L.	Malvaceae	guo mai niu/ban teng (D)	朱槿	SE, S & SW Yunnan		All parts of flower, mostly petal	Flower only	Vegetable	Tea substitute; stewed; roasted; soaked in liquor	Clearing lung-heat and expelling phlegm	0.008	BN165
*Hibiscus sabdariffa* L.	Malvaceae	nui pa song (D)	玫瑰茄	S Yunnan	Cultivated	Calyx	Flower only	Tea substitute, fooddye, beverages	Tea substitute	Whole plant is edible and used as medicine for clearing heat-toxin, lubricating the intestines and relieving the cough. The fibers of the stem bark can be used as a substitute for hemp	0.016	BN124
*Houpoea officinalis* (Rehder & E. H. Wilson) N. H. Xia & C. Y. Wu	Magnoliaceae	Pa zei (Y)	厚朴	W Yunnan	Cultivated	Flower buds	Flower only	Vegetable, tea substitute	Cooked in water; tea substitute	For treating ailments of spleen and stomach, and inappetence	0.004	BN381
*Hydrocharis dubia* (Bl.) Backer	Hydrocharitaceae	Mian rong yang (D)	水鳖	C & S Yunnan	Wild	Peduncle	With young leaves	Vegetable	Stir-fried; cooked in water	Used for clearing heat and promoting diuresis	0.016	BN306
*Hylocereus undatus* (Haw.) Britt. et Rose	Cactaceae	Ba wang hua (YN)	量天尺	S Yunnan	Cultivated	All parts of flower, sometimes petal	Flower only	Vegetable	Cooked in water; stewed	For pulmonary tuberculosis	0.008	BN317
*Hypericum henryi* Lévl. et Van	Hyperiaceae	Zeqna meiqbiei (H)	西南金丝梅	Most areas of Yunnan	Wild	Flower	Flower only	Vegetable	Stir-fried; cooked in water	Used as medicine for treating cold, sore throat, stones, nephritis	0.008	BN272
*Imperata cylindrica* (L.) Beauv	Gramineae	Ha (D), wu ji (H), gang (Y)	白茅	Whole Yunnan		Inflorescence	Flower only	Vegetable	As seasonings; tea substitute	Treating gastric bleeding and nephritis	0.024	BN116
*Ipomoea alba* L.	Convolvulaceae	Chang e ben yue (YN)	月光花	S Yunnan	Wild	Flower	With young leaves	Vegetable	Stir-fried; cooked in water; soup (dry flowers)	Whole plant and seed for medicine	0.008	BN307
*Jasminum sambac* (L.) Aiton	Oleaceae	Nuo chuan, hei luo shuo nai (D)	茉莉花	Whole Yunnan	Cultivated	Flower	Flower only	Vegetable, tea-scenting flower, snack	Scenting tea; stir-fried; roasted	Clearing heat-toxin. Analgesic, soothing, stable, antibacterial, detoxifying, and detumescent	0.024	BN139
*Juglans regia* L	Juglandaceae	Ma dui (D), amei bolhov (H)	核桃花	Whole Yunnan	Cultivated, imported	Male inflorescence	Inflorescence only	Vegetable	After blanching in hot water, stir-fried with pork or others	Walnut is a famous nut. Kernel and bark used as medicine. Timber for sculpture	0.016	BN172
*Justicia adhatoda* L.	Acanthaceae	mo san ber/mo ba heo (D)	鸭嘴花	S Yunnan	wild	Inflorescence	Flower only	Vegetable	Stir-fried;cooked in water	Decoction of roots and leaves for kidney stone, and stomachache	0.016	BN144
*Lablab purpureus* (L.) Sweet	Leguminosae	luo jie (JN)	扁豆	Whole Yunnan	Cultivated	All parts of flower	Flower only	Vegetable	Fried; steamed; cooked in water	Young fruit pods and seeds are edible. Reliving summer heat and removing dampness for regulating stomach	0.008	BN203
*Lagenaria siceraria* (Molina) Standl	Cucurbitaceae	a pu go we/a ga pu we (L), wo pu (JN)	葫芦	Whole Yunnan	Cultivated	All parts of flower	Flower only	Vegetable	Fried; steamed; cooked in water	Fruit is a common vegetable. Anti-inflammatory	0.004	BN336
*Leucocasia gigantea* (Blume) Schott	Araceae	pei heng (D), yu cai/di shui yu (YN)	大野芋	S & SE Yunnan	Cultivated	Peduncle, whole inflorescence	Flower only	Vegetable	After removing the peel of petiole, stewed; steaming with eggplants	Rhizomes for medicine. Leaves can be used as fodder for pigs.Rhizome for removing toxic substance for detumescence, expelling phlegm and resolving convulsion	0.016	BN261
*Limnocharis flava* (L.) Buch	Alismataceae	pa gan gan guo (D)	黄花蔺	S Yunnan	Cultivated	Flower, flower buds	Flower only	Vegetable	Steamed; stir-fried	Aquatic ornamental. Whole plant as forage	0.016	BN099
*Lirianthe coco* (Loureiro) N. H. Xia & C. Y. Wu	Magnoliaceae	Ye he hua (YN)	夜香木兰	S Yunnan	Cultivated	All parts of flower	Flower only	Tea-scenting flower	Scenting tea	Root bark used as medicine to treat headache, liver ailments and rheumatic flaccidity	0.004	BN207
*Litsea cubeba* (Lour.) Pers	Lauraceae	sha hai teng (D), shi bi (H), miu sa pa mi (JN), mu jiang zi (YN)	山鸡椒	Whole Yunnan	Wild, cultivated	All parts of flower, flower buds	Flower only	Seasoning	Stir-fried	Young fruit is a common seasonings. Flower, leaf and fruit peel for perfume oil. Whole plant for dispelling pathogenic wind and cold and regulating qi-flowing for relieving pain	0.016	BN187
*Litsea rubescens* Lec	Lauraceae	Si song maga (L)	红叶木姜子	Most areas of Yunnan	Wild	Flower	Sometimes with young leaves	Vegetable, seasoning	Stir-fried; cooked in water; salad	Used as medicine for treating cold, headache, rheumatism, injuries caused by falls	0.008	BN319
*Lonicera japonica* Thunb	Caprifoliaceae	Dai gao sen/nu dai peng (W), jin yin hua (YN)	忍冬	Whole Yunnan	Cultivated	All parts of flower	Flower only	Vegetable, tea substitute	Tea substitute; cooked in water	Clearing heat-toxin	0.016	BN125
*Luffa acutangula* (L.) Roxb	Cucurbitaceae	ma lai (D)	广东丝瓜	S Yunnan	Cultivated	All parts of flower	Flower only	Vegetable	Fried; steamed; cooked in water	Fruit is a common vegetable	0.004	BN200
*Luffa aegyptiaca* Miller	Cucurbitaceae	ke be chi du we (L), bu lo shi le gai (W), dan lang (D), si kuo (JN)	丝瓜	Whole Yunnan	Cultivated	All parts of flower, male flowers	Flower only	Vegetable	Cooked in water; stewed; stir-fried	Fruit is a common vegetable.Whole plant for medicine. Boiled with eggs for relieving the dizziness. Decoction for throat pain and rhinitis	0.008	BN127
*Melicope pteleifolia* (Champion ex Bentham) T. G. Hartley	Rutaceae	yi xie we (L), lan wang (D)	三桠苦	S & SE Yunnan	Wild	All parts of flower	Flower only	Vegetable	Making congee; cooked in water	Prevention of colds, myelitis, yellow-bile hepatitis, stomach pain, sciatica, etc.	0.004	BN219
*Manihot esculenta* Crantz	Euphorbiaceae	shi qi gu men li we (L), mang niu/a bao (D), la pi (H), a zhe me (JN)	木薯	Tropical Yunnan	Cultivated	All parts of flower	Flower only	Vegetable	Stir-fried; cooked in water	Roots rich in starch. Root and leaf burned for bone fractures	0.008	BN227
*Markhamia stipulata* (Wall.) Seem	Bignoniaceae	ma ya (H), biao bu (JN), dou gan (D)	猫尾木	S & SE Yunnan	Wild	Petal, sometimes all parts of flower	Flower only	Vegetable	Removing satmens and pistils, stir-fried; roasted; cooked in water; salad after blanching in hot water	Young leaves and pods are vegetables and medicine. Leaves for clearing heat-toxin and cooling blood. Roots for clearing heat-toxin	0.016	BN160
*Mayodendron igneum* (Kurz.) Kurz	Bignoniaceae	luo g bi/fa mei lao mei (D), le dou le bo (JN), mo bi we (L), ai dou ma/ma ta ma ye (H), dung lang fang (Y)	火烧花	S & SE Yunnan	Wild, cultivated	All parts of flower, mostly petal	Flower only	Vegetable	After removing calyx and stamens, and blanching in water, stir-fried; cooked in water; salad	Barks for clearing heat-toxin, dispelling pathogenic wind for diuresis and killing ascarid for relieve itching	0.036	BN046
*Melastoma malabathricum* L.	Melastomataceae	Ya gai dian (D), bai na (JN)	印度野牡丹	Tropical Yunnan	Wild	Flower	Flower only	Vegetable, snack	Salad; cooked in water	Ornamental	0.024	BN164
*Michelia* × *alba* DC	Magnoliaceae	zhang hao (D), mian gui hua (YN)	白兰	S Yunnan	Cultivated	All parts of flower	Flower only	Vegetable, tea-scenting flower	Scenting tea; stir-fried	Warming lung for relieving cough and activating qi for resolving turbidity	0.024	BN057
*Michelia champaca* L.	Magnoliaceae	zhang ba leng/mai zhang ha/mai han mu (D)	黄缅桂	S Yunnan	Cultivated	All parts of flower	Flower only	Tea-scenting flower	Scenting tea	Leaves and buds for medicine	0.016	BN222
*Momordica charantia* L.	Cucurbitaceae	ma huai/ma hai kong (D)	苦瓜	Whole Yunnan	Cultivated	Flower	Flower only	Vegetable	Stir-fried; cooked in water	Fruit is a common vegetable. Whole plant for clearing heat-toxin. Fruit for treating diabetes	0.008	BN103
*Monochoria vaginalis* (Burm. f.) Presl ex Kunth	Pontederiaceae	pa geng (D), nuo mu niu pou (H)	鸭舌草	S Yunnan	Wild	All parts of flower	With young stems and leaves	Vegetable	Stir-fried	Whole plant for clearing heat-toxin and diuresis, for anti-inflammition	0.016	BN176
*Moringa oleifera* Lam	Moringaceae	La mu (YN)	辣木	Tropical Yunnan	Cultivated, imported	Inflorescence	Flower only	Vegetable	Stir-fried; cooked in water; soup	All parts are used for food, medicine or other purposes	0.016	BN229
*Morus alba* L.	Moraceae	bei man suai (D), ma sang hua (YN)	桑	Whole Yunnan	Cultivated	Male inflorescence	Inflorescence only	Vegetable	Stir-fried	Clearing lung-heat, relieving rheumatism and nourishing liver and kidney	0.016	BN118
*Mucuna macrocarpa Wall*	Leguminosae	Hei liang long (D), Odmate (L)	大果油麻藤	S Yunnan	Wild	Inflorescence	Flower only	Vegetable	Removing stamens and blanching in hot water, stir-fried; cooked in water	Vine used as medicine for treating anemia, irregular menstruation, rheumatic pain	0.016	BN325
*Murraya exotica* L. Mant	Rutaceae	lon mai shuang ao la (D)	九里香	S & SE Yunnan	Cultivated	All parts of flower	Flower only	Vegetable, tea-scenting flower	Scenting tea	Roots and leaves for medicine	0.008	BN228
*Musa acuminata* Colla	Musaceae	gui teng (D), a bo jie (L), pa lu/a pa la/a se (JN), a ja jao (JP), ye ba jiao hua (YN)	小果野蕉	S & SW Yunnan	Wild	Inflorescence	Inflorescence only	Vegetable	Stewed with pork; stir-fried; cooked in water	Fruits and pseudostems for food or fodder. For bronchitis and dysentery. Moistening lung for removing phlegm, calming liver wind, warming lung for relieving cough, dispersing blood stasis and clearing menstruation	0.086	BN004
*Musa basjoo* Sieb. et Zucc	Musaceae	a du (H), bi (D), ge lou fang (Y), ar du (JN), ba jiao (YN)	芭蕉	Whole Yunnan	Wild, cultivated	Inflorescence	Inflorescence only	Vegetable	After blanching in hot water, stir-fried; cooked in water; roasted; steamed	Fruit is a common local fruit. The leaves can be used as tableware in various ways. Whole plant for clearing heat and diuresis. Dissipating phlegm and resolving masses, suppressing hyperactive liver, dispersing blood stasis and clearing menstruation	0.056	BN068
*Musa* × *paradisiaca* L.	Musaceae	gui (D),a bo jie/a bo we (L), nwadu/a du (JN)	大蕉	S Yunnan	Cultivated	Inflorescence	Inflorescence only	Vegetable	After blanching in hot water, stir-fried; cooked in water	Fruits and young leaves are edible. Pseudostems for fodder. Tubers for starch extraction and wine making. For diabetes	0.024	BN090
*Musella lasiocarpa* (Franch.) C. Y. Wu ex H. W. Li	Musaceae	Nga dou (Y), Di lian hua, Qian ban lian hua (YN)	地涌金莲	C & NW Yunnan	Cultivated	Inflorescence	Inflorescence only	Vegetable	After blancing in hot water, stir-fried; cooked in water; stewed with pork heart	Inflorescence and inner pseudostem used as food. Important forage plant. Flowers and bracts applied externally to stop bleeding and reduce inflammation. Boiled inflorescences for intestinal infections, constipation and gynopathy	0.024	BN179
*Mussaenda hossei* Craib	Rubiaceae	ma di (L), pee ya ni ti/ha la ba ba (H)	红毛玉叶金花	S Yunnan	Wild	Nectar	Flower only	Snack	Salad	Roots and leaves for medicine	0.016	BN220
*Nelumbo nucifera* Gaertn	Nelumbonaceae	guo ruo ou (D)	莲	Whole Yunnan	Cultivated	Petal	Flower only	Vegetable	Cooked in water; fried	Rhizome, seeds and leaves are food and medicine. Clearing heat-toxin, eliminating dampness, promoting blood circulation and hemostasis	0.016	BN119
*Nepeta cataria* L.	Lamiaceae	me u ma/mei ya mang (Y), zhi wo (JN), gon m bo (D), jia ge lao miao (H), jing gai (YN)	荆芥	Whole Yunnan	Cultivated, imported	All parts of flower	Always with tender stems and leaves	Vegetable, seasoning	Salad; stir-fried	Whole plant used for treating cold, headache, measles, rubella, and early sores	0.024	BN169
*Nopalxochia ackermannii* BR. et Rose	Cactaceae	Kong que lan (YN)	令箭荷花	Whole Yunnan	Cultivated	All parts of flower	Flower only	Vegetable	Stir-fried after blanching; cooked in water; soup	Whole plant for ornament	0.008	BN273
*Nymphaea nouchali* Burm. f	Nymphaeaceae	Lan lian hua (YN)	延药睡莲	Tropical Yunnan	Cultivated	Petal	Flower only	Vegetable	Stir-fried; tea substitute	Starch extracted from rhizomes used for food	0.008	BN308
*Ocimum basilicum* L.	Lamiaceae	mu nu we (L), pa you xing (D),zhi wei (JN)	罗勒	Tropical Yunnan	Cultivated or escaped	Inflorescence	Always with young stems and leaves	Vegetable, seasoning	Stir-fried with other food; salad	Whole plant for medicine, reducing fevers and expelling intestinal parasites	0.024	BN109
*Ocimum basilicum* var. *pilosum* (Willd.) Benth	Lamiaceae	mu nu we (L), guan guo (D), puo le (JN)	疏柔毛罗勒		Cultivated or escaped	Inflorescence	Always with tender stems and leaves	Vegetable, seasoning	Stir-fried with other food; salad	Whole plant for medicine	0.024	BN166
*Oenanthe javanica (Bl.) DC*	Apiaceae	Pa an e (D), oguo olou (H)	水芹	Whole Yunnan	Wild	Inflorescence	Always with tender stems and leaves	Vegetable	Stir-fried	Young aerial part used as vegetable. Whole plants for treating rheumatism, neuralgia, hypertension	0.016	BN038
*Opuntia dillenii* (Ker Gawl.) Haw	Cactaceae	pa pu we (L)	仙人掌	S Yunnan	Cultivated	All parts of flower	Flower only	Vegetable	Stewed with eggs or pork	Leaves and fruits are edible. Flowers used to treat rectocele	0.004	BN267
*Orthosiphon aristatus* (Blume) Miq	Lamiaceae	ya nu miao (D), mao xu cao (YN)	肾茶	S Yunnan	Wild, cultivated	Inflorescence	Flower only	Tea substitute	Tea substitute	Flowers used as medicine for diuresis	0.016	BN117
*Oroxylum indicum* (L.) Bentham ex Kurz	Bignoniaceae	lou ga (JN), ga miang fang (Y), qian zhang zhi (YN)	木蝴蝶	S & SE Yunnan	Wild	Petal	Flower only	Vegetable	Stir-fried; cooked in water; salad after blanching in hot water	Young leaves and fruits are edible. Seeds and barks for clearing heat and promoting diuresis	0.036	BN104
*Oxalis corniculata* L.	Oxalidaceae	ji zhu ga (L), song xiang ga(D), a wo a duo(JN)	酢浆草	Whole Yunnan	Wild	Flowers	With leaves	Vegetable	Salad	Leaves and fruits are eaten directly. Used as medicine for stomachache	0.016	BN223
*Panax notoginseng* (Burkill) F. H. Chen ex C. Y. Wu & K. M. Feng	Araliaceae	Ye sanqi (D), san qi hua (YN)	三七	SE Yunnan	Imported	Inflorescence	Flower buds only	Vegetable, tea substitute	Stir-fried with meat; cooked in water; stewed with pork; tea substitute	Famous medicine in China, used for treating many diseases, and improving immunity	0.016	BN069
*Perilla frutescens* (L.) Britt	Lamiaceae	mu nu we (L), jue chum (H), lai ma (JN)	紫苏	Whole Yunnan	Cultivated	All parts of flower	With young leaves	Seasoning	Cooked with other food	Flower buds, stems and leaves used for treating blood deficiency and cold	0.016	BN174
*Pinus yunnanensis* Franch	Pinaceae	to du/to we (L)	云南松	Whole Yunnan	Wild	Pollen	Pollen only	Snack	Making cakes; tea substitute; baked	Moistening heat and lung, benefiting qi, dispelling pathogenic wind for hemostasis	0.008	BN320
*Plumeria rubra* L.	Apocynaceae	muo ke zhang ba (D)	鸡蛋花	Tropical Yunnan	Cultivated	Petal	Flower only	Vegetable, beverages, tea substitute	Tea substitute; fried with eggs; cooked in water; steamed; stewed	Clearing heat and promoting diuresis. Moistening lung for arresting cough	0.024	BN163
*Polygala arillata* Buch.-Ham. ex D. Don	Polygalaceae	Ya nan nen (D), jin que hua (YN)	荷包山桂花	Most areas of Yunnan	Wild	Inflorescence	Flower only	Vegetable	Fried with eggs; stir-fried with other food	Used as medicine to treat neurasthenia, palpitations, amnesia, insomnia, cough, phlegm, bronchitis, cystitis	0.024	BN136
*Polyspora chrysandra* (Cowan) Hu ex B. M. Bartholomew & T. L. Ming	Theaceae	pu mo gu we (L)	黄药大头茶	S Yunnan	Wild	All parts of flower	Flower only	Vegetable	Stir-fried; cooked in water	Clearing heat-toxin	0.004	BN337
*Prunus armeniaca* L.	Rosaceae	ma mang (D), xing hua (YN)	杏	Whole Yunnan	Cultivated	All parts of flower	Flower only	Vegetable	After blanching in hot water, as salad mixed with cucumber	Activating blood circulation and for skin caring	0.004	BN236
*Prunus cerasoides* (D. Don) Sok	Rosaceae	Ying hua (YN)	高盆樱桃	Most areas of Yunnan	Cultivated	Inflorescence	Flower only	Side dish	Decorating dishes; salad	Ornamental	0.004	BN253
*Prunus mume* Siebold & Zucc	Rosaceae	ma fong (D), mei zi (YN)	梅	Whole Yunnan	Wild, cultivated	All parts of flower	Flower only	Vegetable	Cooked in water	Regulating qi-flowing for strengthening spleen	0.008	BN129
*Prunus persica* L.	Rosaceae	a we we (L), ma mang (D), she ye (JN), tao hua (YN)	桃	Whole Yunnan	Cultivated	All parts of flower, petal	Flower only	Vegetable	Cooked with other food	Promoting blood circulation for nourishing heart and lubricating the intestines. For skin caring	0.004	BN312
*Pseuderanthemum polyanthum* (C. B.. Clarke) Merr	Acanthaceae	qi a wu ha ye (D), a bo bie bu (JN), a xia wu ha (H)	多花山壳骨	Tropical Yunnan	Wild, cultivated	All parts of flower	Flower only	Vegetable	Stir-fried; roasted in banana leaves; fried	Roots for hemostasis	0.024	BN073
*Pseudognaphalium affine* (D. Don) Anderberg	Asteraceae	ya muo fie (D), a bao me/mu la lo we (L), mi di zha la (H)	鼠曲草	Whole Yunnan	Wild	All parts of flower	With young stems and leaves	Vegetable	Fried; steamed	Whole plant for expelling phlegm, relieving cough and eliminating dampness	0.024	BN152
*Psidium guajava* L.	Myrtaceae	mangomi we (L), guo mu gui xiang la (D), ma gun (JN)	番石榴	Tropical Yunnan	Cultivated	All parts of flower	Flower only	Vegetable, seasoning	Cooked with other food	Fruit is edible. Leaves and stemskins for medicine	0.008	BN031
*Pueraria montana* var. *lobata* (Willdenow) Maesen & S. M. Almeida ex Sanjappa & Predeep	Leguminosae	qu gu (H)	葛	S Yunnan	Wild	All parts of flower, petal	Flower only	Vegetable, beverages	Stir-fried; tea substitute	Dispel the effects of alcohol	0.008	BN072
*Punica granatum* L.	Lythraceae	mai bu gui huo/ma yong ang (D)	石榴	Tropical Yunnan	Cultivated	All parts of flower, calyx	Flower only	Vegetable, beverages	Stir-fried; cooked in water; tea substitute	Astringing for hemostasis, removing blood stasis, relieving pain and and removing toxic substance	0.036	BN081
*Pyrenaria diospyricarpa* Kurz	Theaceae	pu mo gu we (L)	叶萼核果茶	S Yunnan	Wild	All parts of flower	Flower only	Vegetable, seasoning	Stir-fried with other food	Ornamental	0.004	BN296
*Pyrostegia venusta* (Ker-Gawl.) Miers	Bignoniaceae	Pao zhang hua (YN)	炮仗藤	S Yunnan	Cultivated	All parts of flower, petal	Flower only	Vegetable, tea substitute	After blanching in hot water, stir-fried; cooked in water; tea substitute	Stems and leaves for medicine. Dispelling pathogenic wind, removing dampness, promoting blood circulation for removing blood stasis, moistening lung for arresting cough, clearing heat, relieving sore throat and relieving pain	0.008	BN252
*Pyrus betulifolia Bunge*	Rosaceae	Ma li, yu ru (L)	杜梨	C & S Yunnan	Wild	Flower buds	Flower only	Vegetable	Stir-fried; cooked with salty pork	Clearing the intestines, stoping dysentery, and relieving fatigue	0.016	BN079
*Pyrus pashia* Buch.-Ham. ex D. Don	Rosaceae	geng gai fang (Y)	川梨	Whole Yunnan	Wild	Flower buds	Flower only	Vegetable	Stir-fried; cooked with other food; cooked in water	Used to treat cough, emesis and diarrhea	0.048	BN022
*Pyrus pashia var. kumaoni Stapf*	Rosaceae	geng gai fang (Y)	无毛川梨	S Yunnan	Wild	Flower buds	Flower only	Vegetable	Stir-fried; cooked with salty pork	Used to treat cough, emesis and diarrhea	0.008	BN304
*Pyrus pyrifolia* (Burm. F.) Nakai	Rosaceae	man li se we (L)	沙梨	C & S Yunnan	Cultivated	Flower buds	Flower only	Vegetable, tea substitute, beverages	Steamed; fried; tea substitute	Young leaves can be used as tea substitute. Fruits as medicine for Moistening lung for removing phlegm and arresting cough. Anti-alcohol. For whitening skin	0.016	BN274
*Raphanus sativus* L.	Cruciferae	huo pa bai (D), ho be we (L), bai luo bo (YN)	萝卜	Whole Yunnan	Cultivated	Inflorescence	Always with tender stems and leaves	Vegetable	Stir-fried	Treating urinary system stones, and indigestion ailments	0.016	BN270
*Rhododendron decorum* Franch	Ericaceae	Bo hua (YN)	大白花杜鹃	C & W Yunnan	Imported	Petal	Flower only	Vegetable	After removing stamens & pistils and blanching, stir-fried; cooked with broad bean or pork	Roots and leaves used for activating blood circulation to relieve pain, rheumatic pain, and injuries caused by falls. Ornamental	0.016	BN191
*Rhododendron excellens* Hemsl. et Wils	Ericaceae	Bai hua du juan (YN)	大喇叭杜鹃	S Yunnan	Wild	Petal	Flower only	Vegetable	After removing stamens & pistils and blanching, stir-fried; cooked with broad bean or pork	Ornamental	0.008	BN277
*Robinia pseudoacacia* L.	Leguminosae	Huai hua (YN)	刺槐	C Yunnan	Imported	Inflorescence	Flower only	Vegetable	Salad; stir-fried; cooked in water; fried; steamed; tea substitute	Decoction of inflorescence for spasm, clearing heat, expelling phlegm, lowering blood pressure and diuresis	0.008	BN339
*Rosa banksiae* Ait	Rosaceae	Bai ci hua (YN)	木香花	Whole Yunnan	Cultivated	All parts of flower	Flower only	Tea-scenting flower, snack	Scenting tea	Relieving pain and hemostasis	0.016	BN363
*Rosa chinensis* Jacq	Rosaceae	nuo lang/nuo bi long (D)	月季花	Whole Yunnan	Cultivated	All parts of flower, petal	Flower only	Vegetable, fooddye	Cooked in water; fried; tea substitute	Promoting blood circulation for activating qi-flowing	0.008	BN168
*Rosa* × *gallica* L.	Rosaceae	Mei gui hua (YN)	法国蔷薇	Most areas of Yunnan	Cultivated	Flower	Flower only	Side dish	Decorating dishes; salad	Ornamental	0.004	BN291
*Rosa multiflora* 'Grevillei'	Rosaceae	Yue ji hua (YN)	七姊妹	Most areas of Yunnan	Cultivated	Flower	Flower only	Vegetable	Stir-fried; cooked in water	Ornamental. Young shoots edible. Used as medicine to treat thirst, diarrhea, malaria, wound bleeding	0.004	BN367
*Rosa rugosa* Thunb	Rosaceae	luo mun shang (D)	玫瑰	Whole Yunnan	Cultivated	All parts of flower	Flower only	Tea-scenting flower, snack	Scenting tea; making cakes	Activating qi-flowing, resolving stagnation, reconciling bleed and relieving pain	0.016	BN121
*Rosa rugosa* var. *rosea* Rehder	Rosaceae	luo min shang (D)	红玫瑰	Whole Yunnan	Cultivated	All parts of flower	Flower only	Tea-scenting flower, snack	Scenting tea; making cakes	Activating qi-flowing, resolving stagnation, reconciling bleed and relieving pain	0.008	BN264
*Rotheca serrata* (L.) Steane & Mabb	Lamiaceae	na pe ra ce da (L), guang san ka (D), ni ya/son ba do niu (H)	三对节	Tropical Yunnan	Wild	Inflorescence	Flower only	Vegetable	Roasted in leaves; mixed with roasted and mashed potato	Used as medicine to treat tonsillitis, pharyngitis, rheumatic bone pain, malaria, hepatitis	0.008	BN214
*Rubus ellipticus var. obcordatus* (Franch.) Focke	Rosaceae	she ne (JN)	栽秧泡, 黄刺莓	Whole Yunnan	Wild	All parts of flower	Flower only	Snack	Salad	Edible fruits. Activating qi-flowing, resolving stagnation, removing blood stasis, reliving summer heat and hemostasis. Decoction of flower for hypermenorrhea	0.004	BN321
*Rubus pluribracteatus* L. T. Lu & Boufford	Rosaceae	lao ya bou (JN), lao ya bou (L)	大乌泡	S & SE Yunnan	Wild	All parts of flower	Flower only	Snack	Salad	Edible fruits. Roots for medicine	0.004	BN371
*Sagittaria trifolia* L.	Alismataceae	Reib bago gheab, kuo li (M)	野慈姑	C & S Yunnan	Wild	All parts of flower	Flower only	Vegetable	Stir-fried	Edible juicy tuber. Used to stop bleeding and cough, and reduce swelling and phlegm	0.008	BN205
*Sagittaria trifolia* L. subsp. *leucopetala* Q. F. Wang	Alismataceae	Ci gu (YN)	慈菇花	Most areas of Yunnan	Cultivated	All parts of flower	Flower only	Vegetable	Cooked in water; making congee	Clearing heat-toxin and anti-tumor	0.008	BN361
*Salvia splendens* Ker-Gawler	Lamiaceae	Yi zhong xue (M)	一串红	Most areas of Yunnan	Cultivated	Nectar	Flower only	Snack	Collecting nectars as snack; salad	Whole plant for medicine. Clearing heat-toxin, cooling bleed and detumescence	0.008	BN255
*Senna septemtrionalis* (Viviani) H. S. Irwin & Barneby	Leguminosae	Huai hua mi (YN)	光叶决明	Whole Yunnan	Wild	Flower	Flower only	Vegetable	Stir-fried; cooked in water	Ornamental and forage	0.004	BN275
*Senna siamea* (Lamarck) H. S. Irwin & Barneby	Leguminosae	ma xhe/mai xi li/ge mai xi li (D), hei xin shu (YN)	铁刀木	S Yunnan	Cultivated	Flower, flower buds	With leaves	Vegetable, tea substitute	After blanching in hot water, stir-fried; cooked in water; salad; tea substitute	Trunk for building and fuelwood. Leaves and fruits for medicine to treat insomnia	0.008	BN055
*Sesbania grandiflora* (L.) Pers	Leguminosae	guo nuo gai/nuo jie fei/pa duan long/ge lou gai (D), a ye piu (H)	大花田菁	S Yunnan	Cultivated	All parts of flower	Flower only	Vegetable	Salad; stir-fried; cooked in water; roasted	Barks as astringent. Removing toxic substance and nourishing liver. Extracts can relieve pain and anti-depression	0.036	BN199
*Smilax riparia* A. DC	Smilacaceae	Jiao sed sen (M)	牛尾菜	Whole Yunnan	Wild	Inflorescence	Always with young leaves	Vegetable	Stir-fried	Roots and rhizomes for rheumatic arthritis syndrome, low back pain caused by fatigue, injuries caused by falls, coughing and wheezing	0.008	BN093
*Smilax zeylanica* L.	Smilacaceae	lan bao (D), pa e pa yai (H), qie ga la (JN)	金刚藤	S Yunnan	Wild	Inflorescence	With leaves	Vegetable	Stir-fried	Rhizome used for treating rheumatic waist and leg pain, injuries from falls, scrofula	0.016	BN110
*Solanum americanum* Miller	Solanaceae	pa pie (D), ra hu lu ji (L), ge ni (JN), ku liang cai (YN)	少花龙葵	S Yunnan	Wild	Inflorescence	With stems and leaves	Vegetable	Stir-fried; cooked in water	Whole plant for medicine to clear heat-toxin and induce diuresis for removing edema	0.024	BN067
*Sophora davidii* (Franch.) Skeels	Leguminosae	Ku ci hua (YN)	白刺花	Whole Yunnan	Wild	Flower	Flower only	Vegetable	Stir-fried; cooked in water	Roots and pods for digestion ailments. Flowers for treating throat swelling and pain, and urinary tract stones	0.036	BN084
*Spathodea campanulata* Beauv	Bignoniaceae	Huo yan hua (YN)	火焰树	Tropical Yunnan	Cultivated	Young inflorescence	Flower buds only	Vegetable	After blanching in hot water, stir-fried; cooked in water	Common ornamental in the tropics	0.004	BN225
*Syzygium aromaticum* (L.) Merr. & L.M.Perry	Myrtaceae	Nuo jian (D)	丁香蒲桃	Tropical Yunnan	Cultivated	Flower buds	Flower only	Vegetable, beverages	Stir-fried; cooked in water	For weakness of the spleen and stomach	0.004	BN012
*Syzygium malaccense* (L.) Merr. et Perry	Myrtaceae	Tie mu aje (L)	马六甲蒲桃	S Yunnan	Cultivated	All parts of flower	Flower only	Vegetable	Stir-fried	Edible fruits	0.004	BN171
*Tamarindus indica* L.	Leguminosae	Mu hang (D), qie bie le (JN), suan jiao (YN)	酸豆	Tropical Yunnan	Wild	Flower	Flower only	Vegetable	Stir-fried; cooked in water	Common fruit in the tropics. Used as medicine to treat heat stroke, loss of appetite, infantile malnutrition, pregnancy vomiting, constipation	0.024	BN126
*Telosma cordata* (Burm. f.) Merr	Apocynaceae	Ye xiang teng (YN)	夜来香	S Yunnan	Cultivated	All parts of flower	Flower only	Vegetable	After blanching in hot water, stir-fried	Leaves for medicine	0.024	BN379
*Thespesia lampas* (Cavan.) Dalz. et Gibs	Malvaceae	Tong mian hua (YN)	白脚桐棉	S Yunnan	Wild	All parts of flower	With young leaves and shoots	Vegetable	Stir-fried	Bark fibers for making ropes	0.004	BN217
*Thunbergia grandiflora* (Rottl. ex Willd.) Roxb	Acanthaceae	山牵牛, hu gou le a bu (JN), ha ge lao ma a ye/na mi na li (H), fang men (Y), log long lie (D)	大花山牵牛	S & SE Yunnan	Wild	Petal	Flower only	Vegetable	Stir-fried; roasted in banana leaves	Roots for dispelling pathogenic wind, seeting bone, relieving rigidity of muscles and activating collaterals, promoting blood circulation, promoting blood circulation and strengthening physique	0.048	BN045
*Thunbergia lutea* T. Anders	Acanthaceae	hu gou le a bu (JN)	羽脉山牵牛	S Yunnan	Wild	Petal	Flower only	Vegetable	Roasted in banana leaves	Ornamental	0.016	BN231
*Thysanolaena latifolia* (Roxb. ex Horn.) Honda	Gramineae	Guo xiangsu (D), mai bu lou (JN)	粽叶芦	Tropical Yunnan	Wild	Young inflorescence	Inflorescence only	Vegetable	Stir-fried; cooked in water	Leaves for riping food. Infructescence used to make brooms. Roots for treating Diarrhea, thirst, cough and asthma	0.036	BN048
*Trachycarpus fortunei* (Hook.) H. Wendl	Palmae	Ge guo (D)	棕榈	C & W Yunnan	Cultivated	Young inflorescence	Young spadix only	Vegetable	After blanching in hot water, stir-fried; cooked in water	Roots, leaves and fruits for medicine. Relieving diarrhea, resolving masses and hemostasis. Flowers soaking in boiling water can be used to cure stomach problems	0.036	BN354
*Trevesia palmata* (Roxb.) Vis	Araliaceae	you dang/da wan/dang bie (D), bie da (H), ben gong (Y), tai (JN)	刺通草	S Yunnan	Wild, cultivated	Inflorescence, All parts of flower	With tender leaves	Vegetable	Removing the peel of peduncle, making salad; stir-fried; cooked in water	Young stems and leaves are edible. Leaves for bruises	0.024	BN120
*Tropaeolum majus* L.	Tropaeolaceae	Nuo uo hang (D)	旱金莲	Whole Yunnan	Cultivated	Flower	Flower only	Vegetable	Stir-fried; cooked in water	Ornamental	0.004	BN216
*Vicia faba* L.	Leguminosae	tu bu (D)	蚕豆	Whole Yunnan	Cultivated	All parts of flower	Flower only	Vegetable	Stir-fried	Common edible legume	0.008	BN230
*Urceola rosea* (Hooker & Arnott) D. J. Middleton	Apocynaceae	pa chou lo (JN), song hei (D)	酸叶胶藤	S Yunnan	Wild	All parts of flower	Always with young leaves	Vegetable	Salad	Young leaves and stems can be cooked. Fruit is edible.Whole plant for inducing diuresis for removing edema and relieving pain	0.036	BN215
*Urobotrya latisquama* (Gagnep.) Hiepko	Opiliaceae	Ling wei mu (YN)	尾球木	S Yunnan	Wild	Inflorescence	Always with young leaves	Vegetable	Stir-fried; cooked in water		0.016	BN375
*Wisteria sinensis* (Sims) DC	Leguminosae	Teng hua cai (YN)	紫藤	Most areas of Yunnan	Cultivated	All parts of flower	Flower only	Vegetable, snack	Stir-fried; cooked in water; stewed; baked with eggs; fried; making cakes and soup	Stem bark, flowers and seeds can be used as medicine to treat muscle and bone pain, meridian wind and energy, and rheumatic pain	0.004	BN294
*Woodfordia fruticosa* (L.) Kurz	Lythraceae	don huang/mai don hong (D)	虾子花	S Yunnan	Wild	All parts of flower	Flower only	Vegetable	After blanching, stir-fried	Roots for medicine	0.036	BN254
*Yulania denudata (Desr.) D. L. Fu*	Magnoliaceae	Ying chun hua (YN)	玉兰	Most areas of Yunnan	Cultivated	All parts of flower, petal	Flower only	Vegetable	Stewed	Dispelling pathogenic wind, dispelling cold for resuscitation and dispersing lung qi	0.008	BN113
*Zea mays* L.	Gramineae	kao long luai (D), ar hu (JN), mai(Y), xia du du ju (H)	玉米	Whole Yunnan	Cultivated	Inflorescence	Young inflorescence only	Vegetable	Stir-fired; salad	Worldwide food crop, also for forage and medicine	0.016	BN128
*Zingiber densissimum* S. Q. Tong & Y. M. Xia	Zingiberaceae	mi bo we (L)	多毛姜	S Yunnan	Wild	Inflorescence	Flower only	Vegetable, seasoning	Stir-fired; cooked with other food	Rhizome used as medicine	0.004	BN316
*Zingiber officinale* Roscoe	Zingiberaceae	mi bo we (L), wang/king long (D), tao zhi (H)	姜	Whole Yunnan	Cultivated	Inflorescence	Flower only	Vegetable, seasoning	Stir-fired; cooked with other food	Common spice crop and medicine	0.036	BN122
*Zingiber striolatum* Diels	Zingiberaceae	Ye jiang hua (YN)	阳荷	C, SW & S Yunnan	Cultivated	Inflorescence	Flower only	Vegetable	Stir-fried; cooked in water; pickled	Bud for medicine	0.036	BN049

Of all the recorded EF species, Zingiberaceae was the largest family with 17 species (8.0%), followed by Leguminosae and Rosaceae each with 16 taxa (7.5%). *Brassica* was the largest genera with 8 taxa (3.8%). Most EFs were cultivated or can be cultivated (127 species), more than wild (83), imported (8) and escaped (5) ones.

**Fig. 2:**
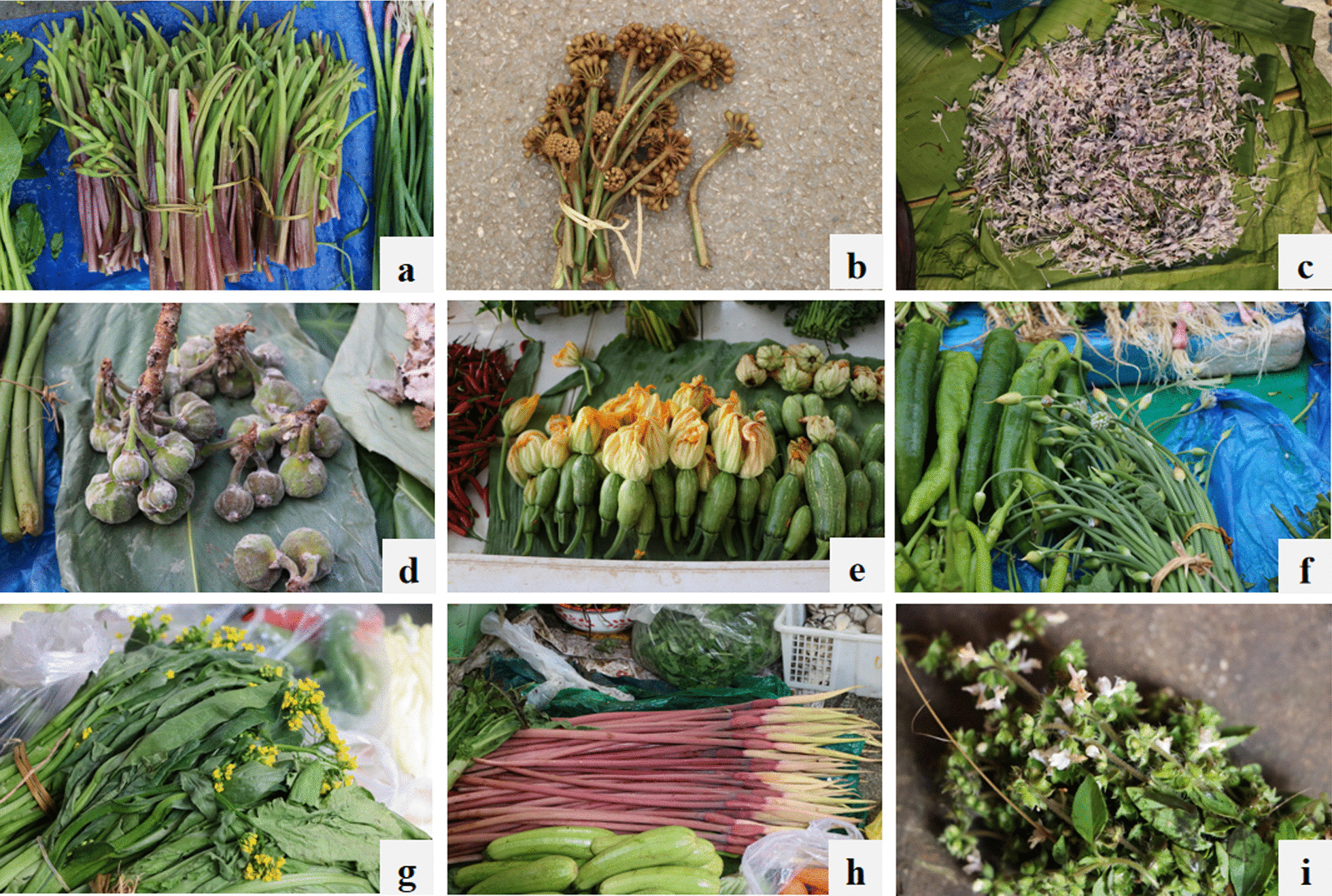
EFs used as vegetables and seasonings. **a** *Limnocharis flava* (L.) Buch.; **b** *Trevesia palmata* (Roxb.) Vis.; **c** *Pseuderanthemum polyanthum* (C.B.Clarke) Merr.; **d** *Ficus auriculata* Lour. (hypanthium); **e** *Cucurbita moschata* (Duch. ex Lam.) Duch. ex Poiret (female flowers); **f** *Allium tuberosum* Rottler ex Sprengle; **g** *Brassica rapa* var. *glabra* Regel (with leaves and stems); **h** *Colocasia esculenta* (L.) Schott. **i** *Nepeta cataria* L. (with leaves and stems)

There were 121 taxa (57.1%) distributed in the south of Yunnan or tropical Yunnan, such as *Limnocharis flava*, *Trevesia palmata*, *Pseuderanthemum polyanthum* and *Ficus auriculata* (Fig. 2a–d). A total of 80 EFs (37.7%) can be found in the Yunnan or most areas of Yunnan, showing the wide flower-eating custom or culture in Yunnan province. For example, the flowers of *Cucurbita moschata* (both male and female flowers, mostly male flowers consumed only), *Brassica rapa* var. *glabra* and *Colocasia esculenta* are very common and widespread as vegetables (Fig. 2e, g, h). The inflorescence of *Allium tuberosum* (Fig. 2f) and *Nepeta cataria* (Fig. 2i) are always used as seasonings in various stir-fries and grilled fish throughout Yunnan.

### Cultural diversity of edible flowers

**Fig. 3:**
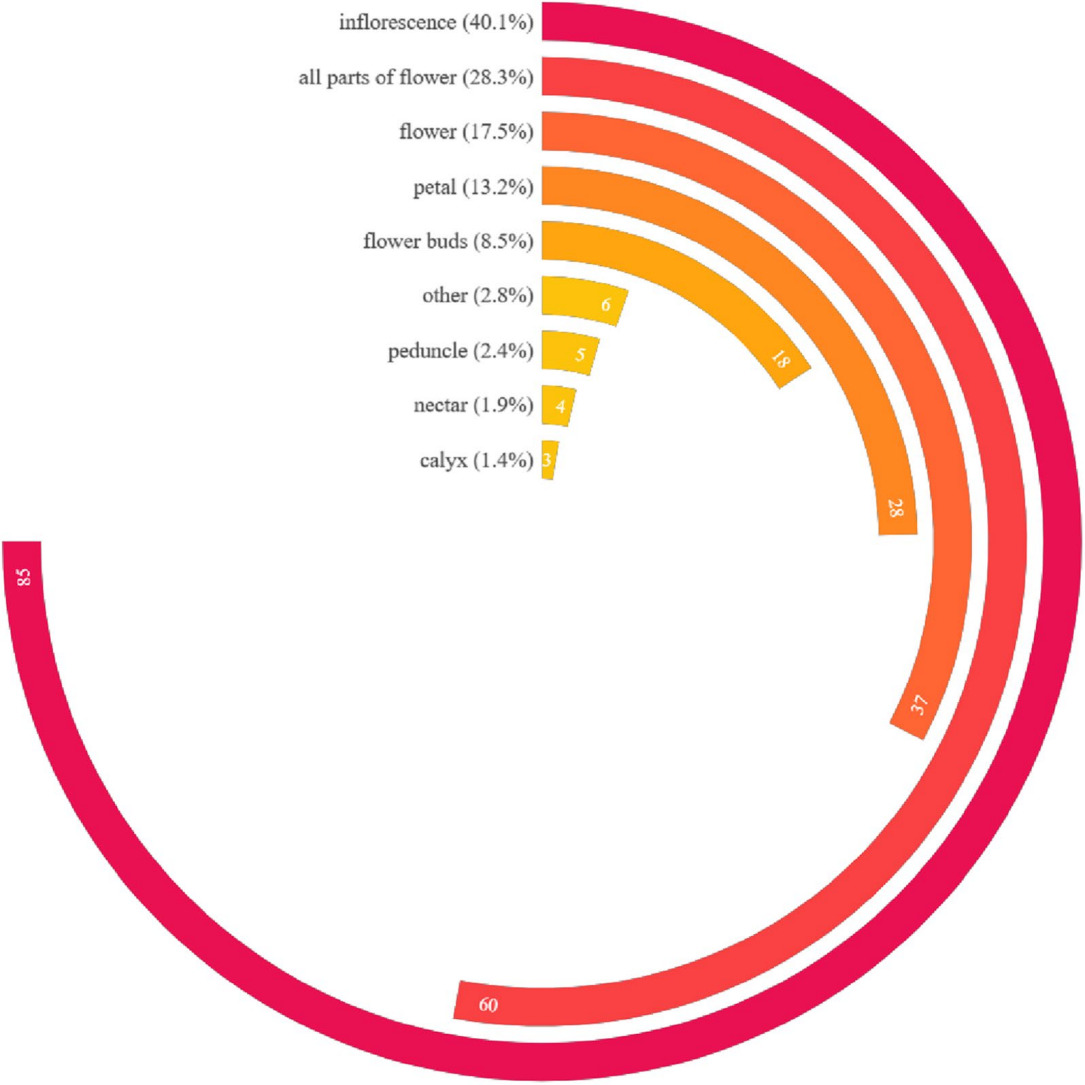
The diversity of flower parts used for food. The number refers to the species number, and the percentage refers to the ratio of each category of edible part of flowers

The edible parts of the EFs used for food in Xishuangbanna were various. These include all parts of the flower, inflorescence, flower (entire flower as a whole), petals, flower buds, nectar, peduncle, calyx, pollen, receptacle, stamens, spathes and scape (Fig. 3). Consuming an entire flower as a whole and consuming specific parts of the flower are distinct concepts. When referring to eating all parts of the flower, it implies that the different components of the flower organ can be consumed individually. When consuming flowers of *Bombax ceiba*, for example, its fresh stamens are stir-fried as dishes and its petals are cooked to make soups, while the dry flowers are used in herbal tea. The “flower” means that the whole flower is eaten together without separating the different parts of the flower organ. Through our investigation and statistics, the most consumed part was inflorescence as a whole (85 species, 40.1%), followed by all parts of flower (60, 28.3%). A total of 181 EFs (85.4%) exhibit the presence of multiple edible parts. This finding highlights the profound knowledge and extensive utilization of local plant resources by the indigenous population.

**Fig. 4:**
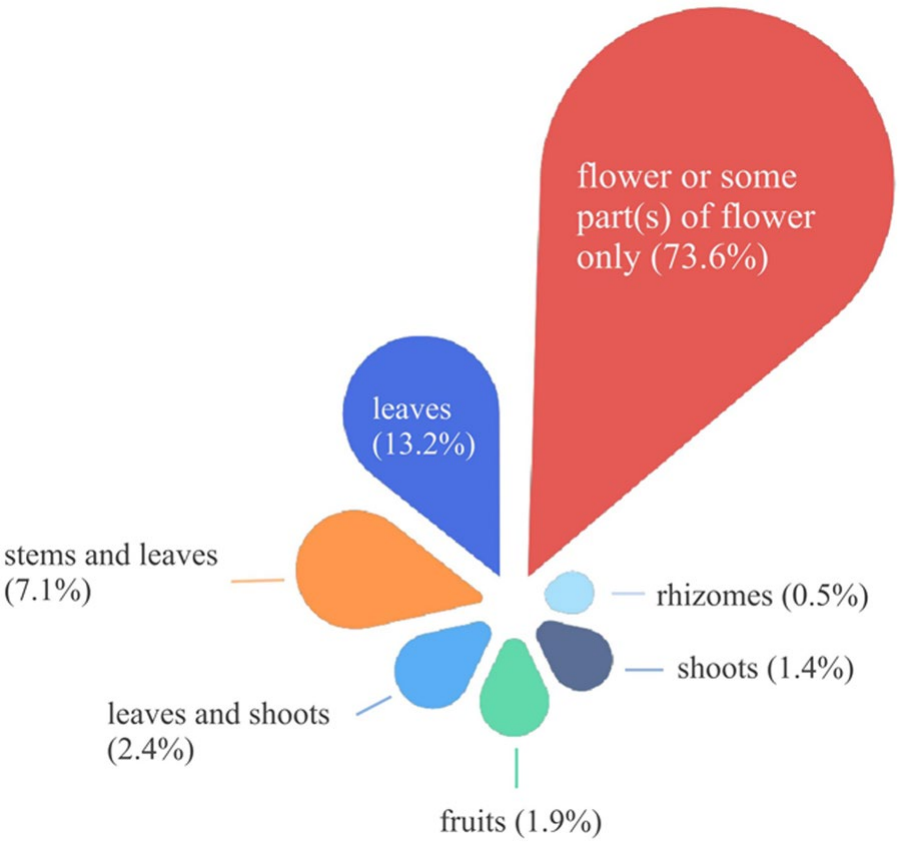
The diversity of other edible parts when collecting/ consuming flowers. The percentage refers to the ratio in all edible flower species

Mostly, flowers for eating were collected or used alone (156 species, 73.6%). They were also used with other parts of the plant, being collected or consumed with leaves, stems, rhizomes, fruits, shoots and pods (Fig. 4). Leaves were the most often consumed together with flowers (43 species, 20.3%). Flowers from *Brassica* plants (*Brassica juncea*, *B. oleracea*, *B. rapa* var. *chinensis*, *B. rapa* var. *glabra*, and *B. rapa* var. *oleifera*), *Ocimum basilicum*, *Nepeta cataria*, and *Solanum americanum* were always gathered and consumed together with their stems and leaves. They were common in the Xishuangbanna markets and are well-known by locals with higher RFC values. Interestingly, when we conducted the semi-structured interviews in the villages, respondents would never mention them unless we initially asked if they were EFs first. In locals’ perceptions, these EFs are a little different from other leafy vegetables because they were always commonly cultivated and had small flowers and limited cultural significance.

**Fig. 5:**
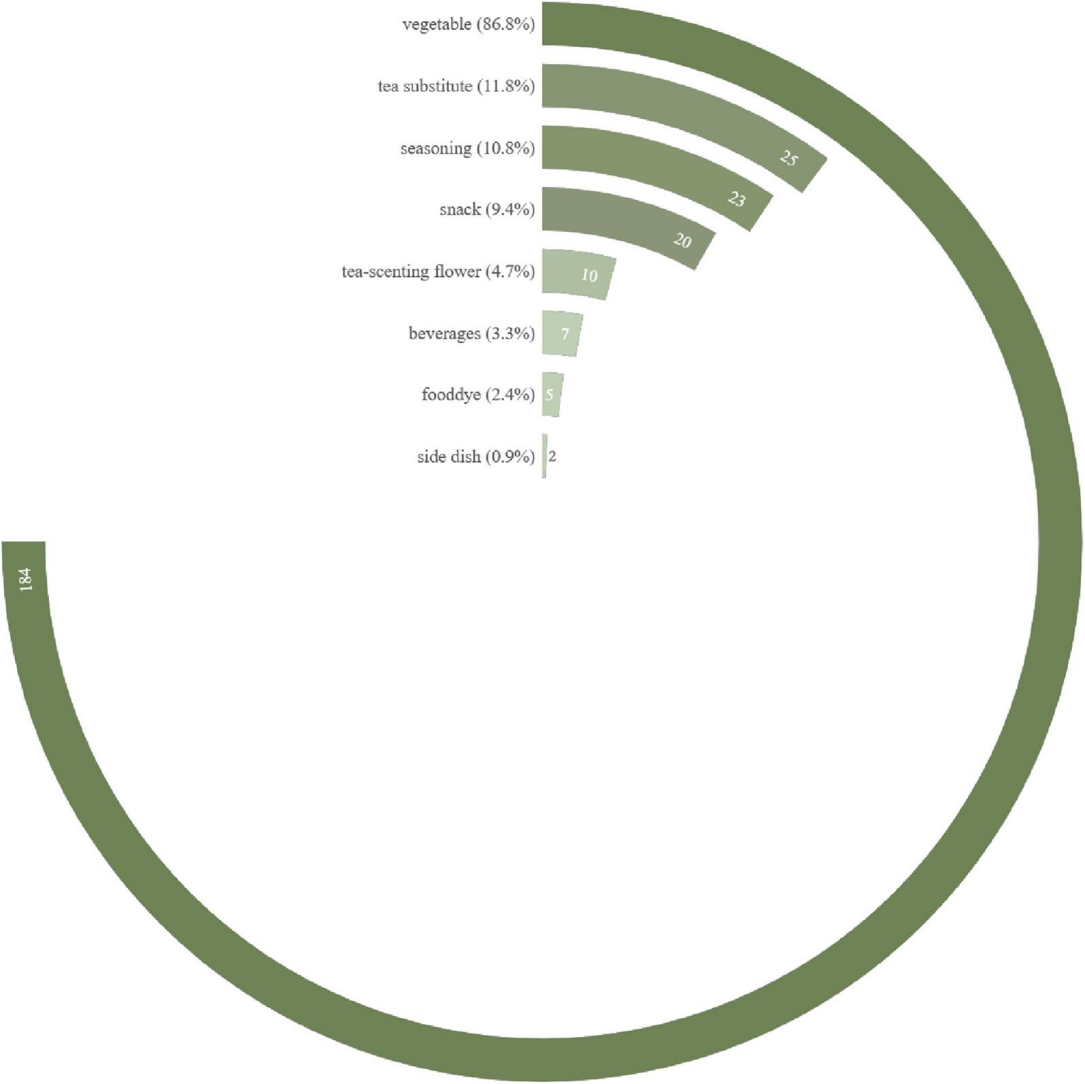
The diversity of use type of EFs. The number refers to species number, and the percentage refers to the ratio in all edible flower species

All EFs species were also classified by their use types. In Xishuangbanna, locals used EFs as a vegetable, tea-scented flower, tea substitute, seasoning, snack, food dye, beverage, and side dish (Fig. 5). Vegetables were the most common product type with 184 EFs (86.8%). It was also common to use EFs as seasonings, tea substitutes and snacks. The majority of EFs (25.9%) have more than one use type.

**Fig. 6:**
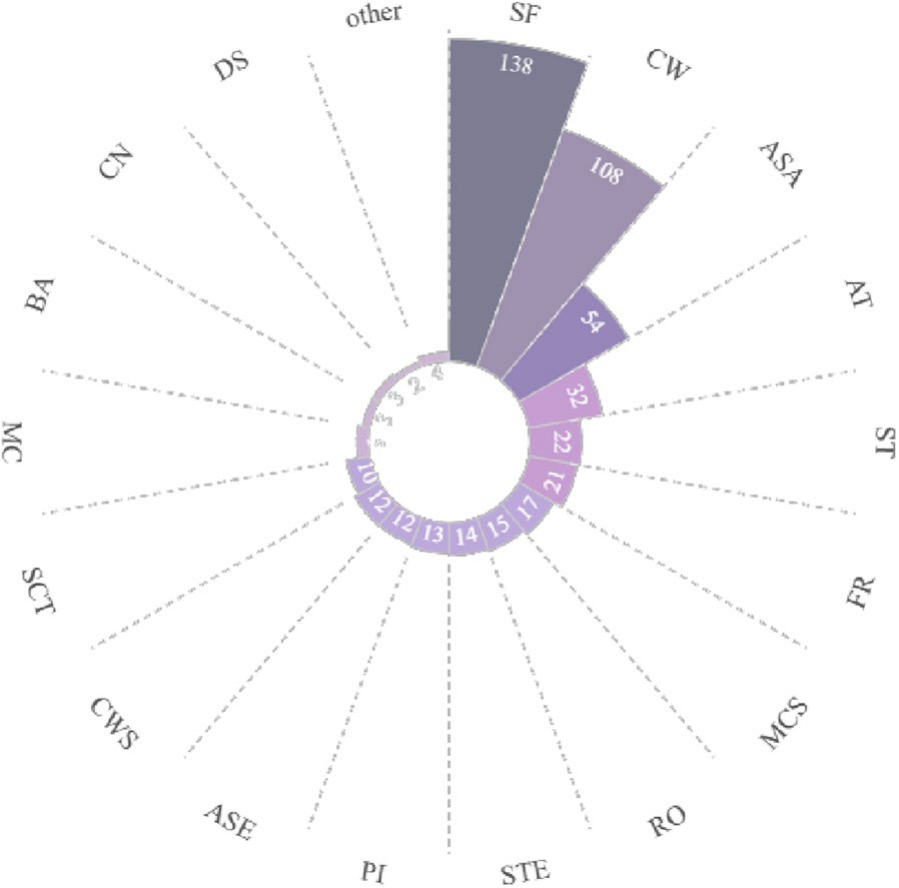
The diversity of methods to prepare EF food (SF: stir-fried; CW: cooked in water; ASA: as a salad; AT: as tea substitute; ST: stewed; FR: fried; MCS: making congee or soup; RO: roasted; STE: steamed; PI: pickled; ASE: as seasonings; CWS: cooked with something; SCT: scenting tea; MC: making cakes; BA: baked; CN: collecting nectars as snack or sweetener; DS: dying something)

Due to different EFs and dietary cultures, food made from EFs was prepared using a variety of methods, including stir-frying, cooking in water, steaming, stewing, pickling, frying, roasting, baking, brewing, cooking with something, dying something, scenting tea, collecting nectars as snack, making congee, soup or cakes and as salad, seasonings or tea substitute (Fig. 6). Stir-frying (138, 65.1%) was the most common way to prepare these flowers, followed by cooking in water (108, 50.9%). The term “other” includes brewing, soaking in liquor, cooking as a vegetable in a hot-pot, and making sweets.

Furthermore, 29 species of edible flowers (EFs) (13.7%) necessitated boiling, blanching in hot water, or the removal of certain parts such as peels and stamens prior to cooking and consumption. Among these, *Mayodendron igneum* is a notable EF long utilized in Xishuangbanna and engaged in games by the Jinuo community (Fig. 8b). Children from the Jinuo group delight in blowing into its tubular corolla, reminiscent of an orange trumpet. To prepare the flowers for food, the calyx and stamens were removed and then, blanched in water to eliminate bitterness. They can then be stir-fried, boiled, or used as a salad. Some slightly toxic EFs, such as *Rhododendron* flowers, also required pre-treatment to reduce their toxicity. *Colocasia esculenta* is a very common EF in Yunnan. Before consumption, people remove the peel of the petiole and spadix from *C. esculenta*. Even so, some people may experience mild discomfort, such as numbness of the tongue or a sore throat. However, rest assured that it is safe for consumption in this manner.

### Multiple uses of flower-eating plants

**Fig. 7:**
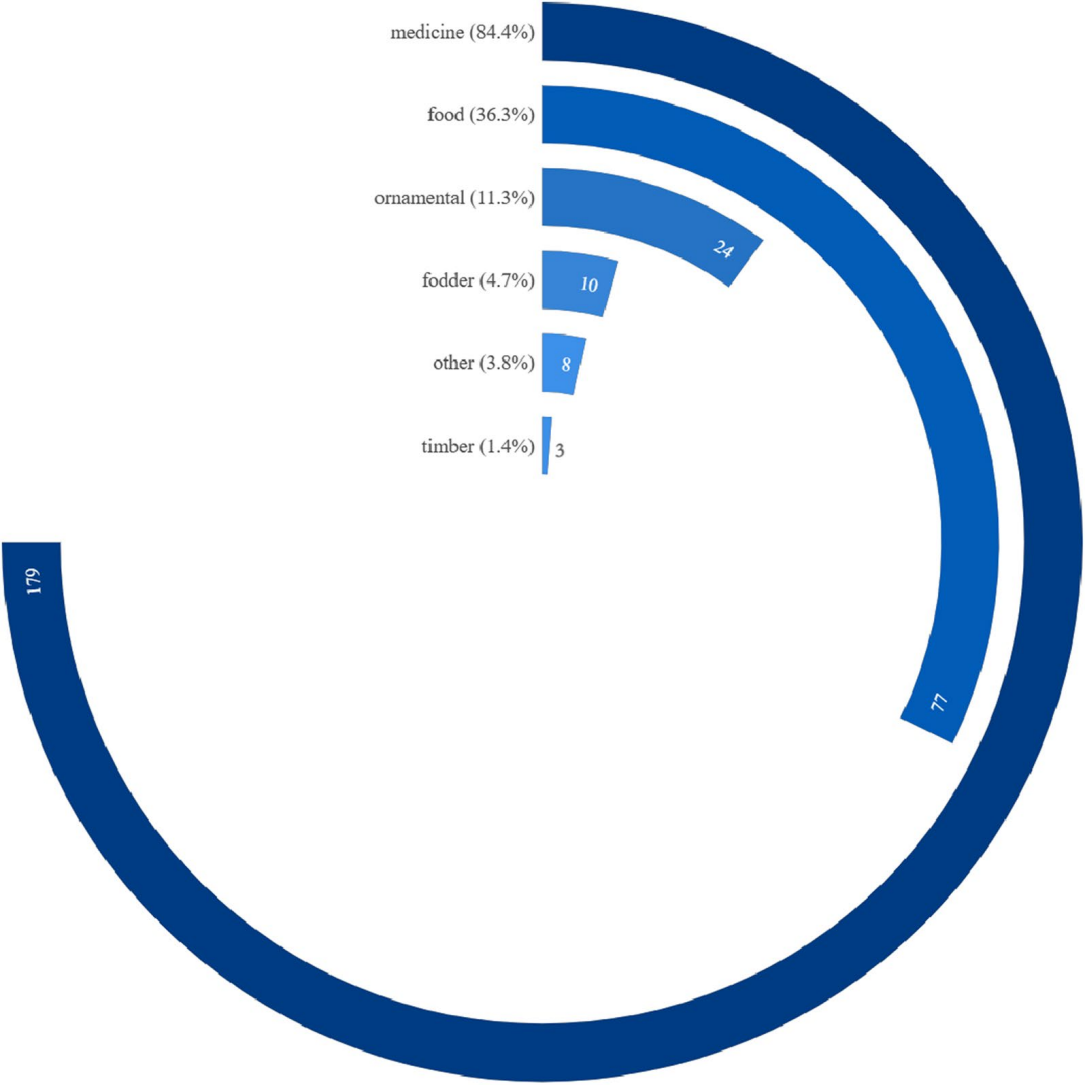
Additional uses of flower-eating plants. The number refers to species number, and the percentage refers to the ratio in all edible flower species

All EFs have multiple uses beyond food. A total of 179 EF species (84.4%) showed medicinal values (Fig. 7). Apart from flowers, other parts of some species, such as roots, stems, leaves, fruits and seeds, can be used for a variety of purposes. These include clearing heat, invigorating the stomach and promoting blood circulation.

Flower-eating plants were also used as ornamentals, fodder, and timber (Fig. 7). The pseudostem of *Musella lasiocarpa* was edible, and it was an important feed for local pigs. *Musa acuminata* leaves were commonly used for containing food and as tableware in various ways. Kernel of *Prunus armeniaca* can be used for skincare. The bark fibers of *Thespesia lampas* were utilized for producing ropes and the seed hairs of *Bombax ceiba* for beddings. The chopping boards made from the trunk of *Senna siamea* were sold in many markets. EF resources affect every aspect of people’s lives in Xishuangbanna.

### EFs with significance in a traditional culture based on RFC values

**Fig. 8:**
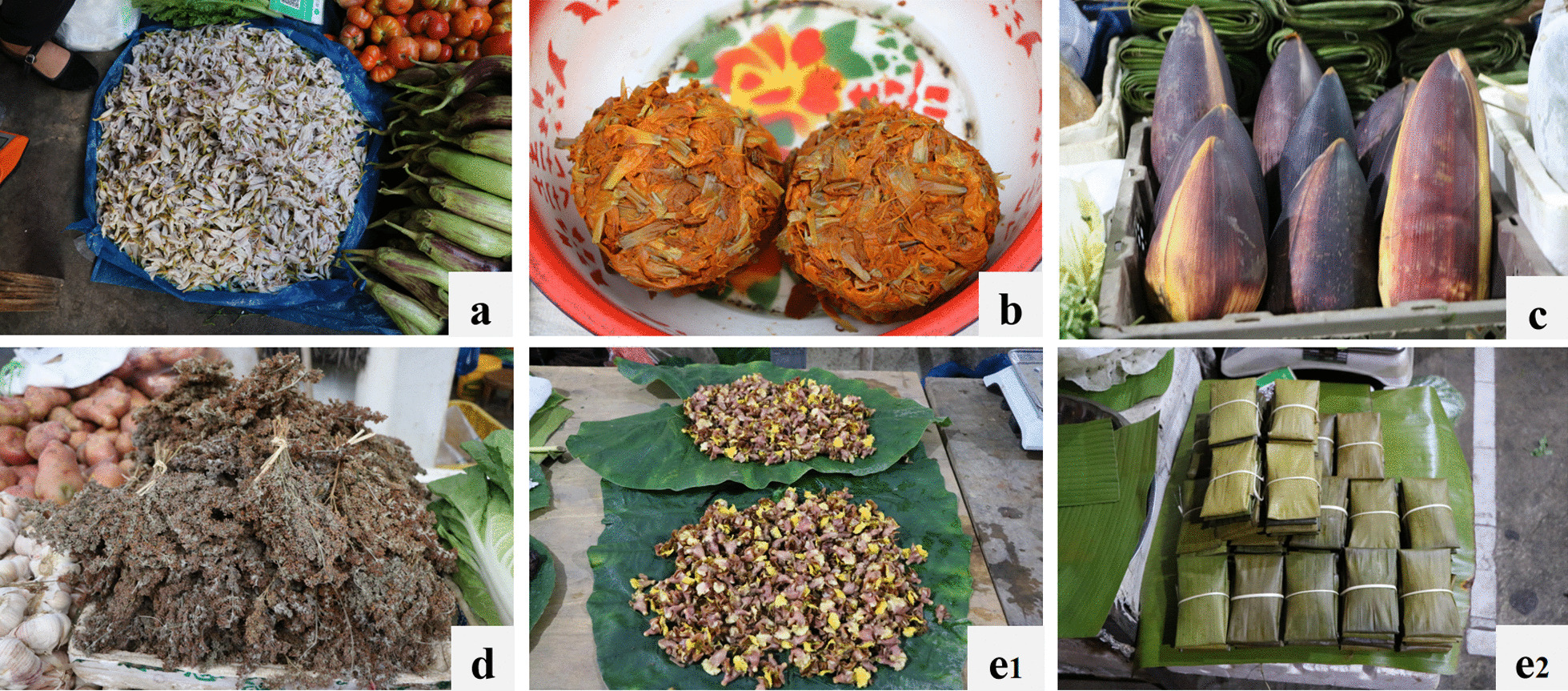
Some EFs with significance cultural values in Xishuangbanna (**a** *Bauhinia variegata* var. *candida* (Roxb.) Voigt; **b** *Mayodendron igneum* (Kurz.) Kurz.; **c** *Musa acuminata* Colla; **d** *Buddleja officinalis* Maxim.; **e1** & **e2**: *Gmelina arborea* Roxb.)

The RFC values of each EFs were calculated to reflect their local importance. *Bauhinia variegata* var. *candida*, *Brassica oleracea* var. *botrytis* and *Musa acuminata* have the highest RFC values. While *Brassica oleracea* var. *botrytis* is renowned worldwide and widely used in China, it holds no special meanings in Xishuangbanna. *Musa acuminata* is a common and distinctive vegetable frequently consumed by the locals (Fig. 8c). The plant’s inflorescence, in particular its bracts and petals, can be utilized as a culinary ingredient in various dishes. It can be prepared by stewing or stir-frying with pork, roasting, steaming or boiling in water. The Yao people in Yiwu Town, Mengla County, have a tradition to celebrate a Chinese festival called Long-tai-tou, the Dragon-Raising-Head Festival. During the festival, the whole community eats wild banana flowers (inflorescence of *Musa acuminata*) so that they would have a good harvest year-around. Traditionally, the flower is stir-fried with pork and then, wrapped in glutinous rice, making it a delectable festival delicacy.

*Bauhinia variegata* var. *candida* is a common species in the south of Yunnan. The flowers (Fig. 8a), young leaves and pods were consumed as vegetables in Xishuangbanna. After blanching in hot water, the flowers can be stir-fried, cooked in water, fried, salad or stewed. The ethnic groups, particularly the Jinuo people in Xishuangbanna, have a long history of using the flower of *Bauhinia variegata* var. *candida*, which commonly known as “*jiebo*.” Living in the mountains and traditionally practicing slash-and-burn agriculture, the Jinuo people consume its flowers along with this young leaves. Its tender fruits (pods) can be preserved and eaten for up to two months. The *jiebo* flower serves as an indicator species for farming and holds cultural importance for the *Jinuo* farming. The Jinuo’s old saying “When the *jiebo* blooms and the cicada chirps, it is time to sow seeds” indicates the significance of *Bauhinia* flower in agricultural activities. The *jiebo* flower holds a social significance among the youths, symbolizing a period of heightened social interactions when in full bloom. Climbing higher up the tree to gather more *jiebo* flowers is seen as a demonstration of capability and becomes an attractive trait to young girls.

*Buddleja officinali*s and *Gmelina arborea* flowers possess the third-highest RFC values, signifying their important roles in flower-eating culture in Xishuangbanna (Fig. 8d, e1). *B. officinalis* was frequently employed as a food dye. Its inflorescences are sun-dried, and during rice preparation, they were immersed in hot water until the water takes on a yellow hue. Following this, glutinous rice was soaked in the water containing the *Buddleja* pigment for 4–5 h. Subsequently, the water was filtered, and the rice was steamed using a wooden pot. The golden yellow rice is then made with a subtle fragrance, which is a traditional festival food on March 3^rd^ of the Chinese Lunar Calendar. The inflorescence in boiled water can also be utilized as tea substitute. The cake made from *G. arborea* flower was very common and can be easily found in markets (Fig. 8e2).

## Discussion

### Flower-eating culture as a biocultural contribution to biodiversity conservation

The practice of consuming flowers is not confined to a particular region or linguistic group; rather, it’s a widespread human custom [10]. Nevertheless, the species consumed, the motivations, and the distinct behaviors related to flower consumption can vary across cultures [19]. Among the most common 15 species worldwide counted by Lu et al., there are only 8 species included in Table 2, implying the difference between EF species in Xishuangbanna and other places in the world [10]. The significant difference is due to abundant local biodiversity and deeply rooted traditional culture in Xishuangbanna, which represents rich biocultural diversity [40].

Cultural heritage and biological diversity share an intricate bond, with the associated knowledge being equally crucial alongside the diverse array of the EFs [41]. Thorough documentation of the local traditional knowledge holds a vital role in safeguarding biocultural diversity [42]. Within Xishuangbanna, the traditional knowledge regarding the usage of the EFs stands as a central and representative component of the flower-eating culture. This knowledge encompasses an elaborate range of practices. That means 13 categories of flower parts can be prepared in 21 distinct methods, yielding 8 distinct types of food. The local community possesses a clear understanding of which EFs require specific processing before cooking to eliminate toxicity or bitterness. They also understand which ones can be harmoniously cooked with other plant parts such as stems and leaves.

Edible plants are part of the biocultural resources that ethnic groups possess [43]. EFs with cultural identities are also part of the local culture or plant culture [44]. The phenomenon of consuming flowers serves the purpose of fulfilling individuals’ physiological requirements for sustenance. It also carries emotional and spiritual values that vary due to diverse cultural communities. The dietary habits within such a wide group and the cultural values of eating specific EF species on specific calendar promote the conservation and sustainable use of these species. Therefore, the preservation and sustenance of biodiversity are ensured.

### Food and medicine continuum links tradition and modern application

In the past, EFs were consumed due to their perceived medicinal attributes [16]. Flowers have served as effective agents in traditional Chinese medicine and ethnomedicine, aiding in matters of beauty, blood circulation, mood modulation, and even sleep enhancement [45]. The *Compendium of Materia Medica*, a comprehensive collection of 52 volumes of materia medica up to the sixteenth century, includes 26 volumes of botanical medicines, of which more than 80 types are flower medicines, accounting for one-tenth of all botanical medicines [46]. In the 2020 edition of Chinese Pharmacopoeia (Volume I), there are 27 flower drugs with the utilizable parts including bud, stigma, inflorescence, receptacle, pollen and intact flower. They are effective at clearing heat, stopping bleeding and promoting blood circulation for removing blood stasis. They have pharmacological effects in the clinical treatment of skin diseases, gynecological diseases, psychiatric diseases and cardiovascular diseases [47]. We also investigated some of the flowers sold in the Xishuangbanna market for medicinal purposes only. Such flowers include *Prunella vulgaris* and *Leonurus japonicus*. They are commonly used as medicine by the local people, but we excluded them from Table 2.

The homology of medicine and food is a part of the traditional culture of various linguistic groups in Xishuangbanna. A total of 95 species belonging to 43 families were identified and summarized [28]. In this study, 179 EFs (84.4%) with medicinal functions were surveyed, much more than other functions. In Xishuangbanna, people possess an extensive range of knowledge concerning medicinal and edible plants, marked by unique ethnic and regional attributes. This knowledge plays a significant role in their daily lives.

The interconnection between food and medicine is a prevalent occurrence observed globally, rooted in ancient cultural practices. The traditional knowledge and wisdom of the food and medicine continuum were critical for people in the past to improve health and defeat diseases. Nowadays, they can provide raw materials and relevant information for healthy food and drugs [48]. The list of substances traditionally used as both food and herbal medicine published by the Chinese authority covers 109 food-medicine dual-use entities, 15 of which are derived from flowers [49]. Most of the medicinal and dietary plants documented in this study are not included, which indicates the potential to scout and research them. The medicine and food homologous flowers link traditional culture and modern applications, deserving much attention and far-reaching research.

### Edible flowers for sustainable development in the future

The utilization of flowers as food is not a recent revelation, yet it often goes unnoticed. Conventionally, flowers, predominantly appreciated for their ornamental appeal, are typically employed for decorative or landscaping purposes in the majority of people's perception [8, 14, 50]. Even in catering, flowers serve to enhance sensory pleasure through their appearances and aroma, though they are often perceived as inedible [9]. In China, the culture of flowers is characterized by the ornamental function and various beautiful implications [51]. Eating flowers is always on the edge of the culture and food system, existing in the rural and indigenous foodscape in China [52]. Among the urban populations, the concept of flower-eating culture is relatively novel, drawing attention due to its unique ability to provide both nourishment and spiritual enrichment to humanity. It also offers a fresh perspective in contrast to consuming leafy vegetables, presenting the potential to contribute to a health-conscious way of living.

The nutritional, phytochemical and pharmacological studies on some representative EFs in Xishuangbanna partly justify the use of EFs by local people and reveal their potential to be exploited. The flower of *Gmelina arborea* is rich in nutrition with various amino acids, minerals, sugars, fiber and trace elements such as selenium. It also has excellent hypoglycemic and antibacterial activity [53]. Pigment extracted from it has good properties and is non-toxic and odorless [54]. *Musa acuminata* inflorescence has high total phenolic content with significant free radical scavenging activity and good antioxidant capacities [55]. *Buddleja officinali*s inflorescence contains flavonoids, phenylethanols, terpenoids, alkaloids and volatile oils, which have a variety of pharmacological activities such as antioxidant, antibacterial, anti-inflammatory, neuroprotective and immunomodulatory [56]. The cake made from *G. arborea* flowers was a widespread delicacy, easily accessible in markets. It held great significance as a staple during the Dai New Year celebration, known as the water splashing festival. During its preparation, Dai women combined dried flower powder (referred to as Maisuo in Dai language) with glutinous rice powder, brown sugar or white sugar, sesame seeds, peanuts, and vegetable oil. The amalgamation was then shaped into cubes, enveloped in banana or *Phrynium* leaves, and steamed in a wooden pot. This steamed cake, called Khaonuosuo by the locals, could be stored at room temperature for around a week due to the bioactive properties of Maisuo, which exhibits antibacterial effects [38, 39].

In fact, studies on the factors influencing consumers’ attitudes toward the EFs’ consumption showed that health benefits are not the most important reason why people consumed EFs [57]. The cultural factors and specific curiosity have great influences on attitudes toward the consumption of EFs according to the survey in three different countries (Portugal, Slovenia, and Brazil), respectively [57], and in Taiwan of China [58]. Whatever the motivation, it is no doubt that there is a huge market for flower-eating to be explored. A questionnaire survey conducted in Portugal, where there is little flower-eating culture, showed that flowers can be popular in gastronomy [59]. As an unfamiliar food in South America, consumers showed a positive attitude to food with flowers either for eating or for decoration [60].

The local farmers’ markets as well as supermarkets provide EFs and derivative products as bridges linking EFs with consumers. The consumption of flowers holds significant importance in the diet of individuals residing in Xishuangbanna. This flower-centric culinary culture has consequently led to the development of associated products. Within local supermarkets, canned pork products are specifically labeled as 'suitable for preparing *Musa* inflorescence' on their packaging. The potential of the EF market lies not only in the development of food and drug products based directly on EFs as materials. It lies also in the creation of more derivative products in the whole EFs knowledge system based on a common perception and a wide range of users.

Though the EFs market has become increasingly promising, toxicity treatment is an issue to be raised [9]. Various toxic and anti-nutritional constituents, including trypsin inhibitors, hemagglutinins, oxalic acid, cyanogenic glycosides, and alkaloids, have been detected in the floral structures[8]. In this study, 29 EFs (13.7%) should be pre-processed by boiling or blanching in hot water, or removing peels and some parts before cooking and eating. Although the locals do not know the exact toxic ingredients, they have traditional knowledge to ensure their safety. Toxicological studies on these EFs can identify specific toxic components or discover novel toxic components that will contribute to consumption safety.

Chinese food is now shifting from traditional cuisine toward more meat and processed foods [61]. It is time to pay attention to healthy eating including the plant-based dietary trends worldwide [62]. Research has established the advantages of plant-based diets for China’s aging population [63]. Combining plant-based food with local production would be an advantageous approach [64], suggesting that EFs could play a substantial role in enhancing the local food system and providing potential solutions to societal challenges such as aging and health-related concerns.

## Conclusion

Through the ethnobotanical survey, 212 species and varieties of edible flowers from 58 families along with a variety of related traditional knowledge in Xishuangbanna were recorded and summarized. Local people are knowledgeable about which EFs are safe when consuming, how to process them by removing toxicity or bitterness, and whether they can be cooked with other parts of the plant. The traditional knowledge on usage of EFs is the main and representative element of flower-eating culture, but it is gradually diminishing. The preservation and further scouting of traditional knowledge of edible and medicinal flowers are critical because of their biocultural significance and great potential for markets, scientific research and industrial exploitation. The practice of consuming flowers, rooted in local traditions, serves as a bridge between tradition and modernity. This flower-eating culture has the potential to promote the conservation of biocultural diversity, healthier food systems and sustainable development in the future. As such, policymakers are able to consider initiatives to support the documentation, conservation, and revitalization of this cultural heritage, recognizing its potential benefits for both local community and broader societal contexts.

## Data Availability

All data generated or analyzed during this study were included in this published article (along with the supplementary files).

## Abbreviations

EF: Edible flower
RFC: Relative frequency of citation

## References

[1] Díaz S, Pascual U, Stenseke M, Martín-López B, Watson RT, Molnár Z, Hill R, Chan KMA, Baste IA, Brauman KA, Polasky S, Church A, Lonsdale M, Larigauderie A, Leadley PW, van Oudenhoven APE, van der Plaat F, Schröter M, Lavorel S, Aumeeruddy-Thomas Y, Bukvareva E, Davies K, Demissew S, Erpul G, Failler P, Guerra CA, Hewitt CL, Keune H, Lindley S, Shirayama Y (2018). Assessing nature’s contributions to people. Science.

[2] Molina-Venegas R, Rodriguez MA, Pardo-de-Santayana M, Mabberley DJ (2021). A global database of plant services for humankind. PLoS ONE.

[3] Zocchi DM, Mattalia G, Aziz MA, Kalle R, Fontefrancesco MF, Sõukand R, Pieroni A (2023). Searching for Germane questions in the ethnobiology of food scouting. J Ethnobiol.

[4] Gori B, Ulian T, Bernal HY, Diazgranados M (2022). Understanding the diversity and biogeography of Colombian edible plants. Sci Rep.

[5] Motti R, Paura B, Cozzolino A, Falco BD (2022). Edible flowers used in some countries of the Mediterranean basin: an ethnobotanical overview. Plants.

[6] Khomdram SD, Fanai L, Yumkham SD (2019). Local knowledge of edible flowers used in Mizoram. Indian J Tradit Know.

[7] Mulík S, Ozuna C (2020). Mexican edible flowers: cultural background, traditional culinary uses, and potential health benefits. Int J Gastron Food Sci.

[8] Purohit SR, Rana SS, Idrishi R, Sharma V, Ghosh P (2021). A review on nutritional, bioactive, toxicological properties and preservation of edible flowers. Future Foods.

[9] Guine RPF, Florenca SG, Ferrao AC, Correia PMR (2019). Investigation about the consumption of edible flowers in Portugal. Indian J Tradit Know.

[10] Lu BY, Li MQ, Yin R (2016). Phytochemical content, health benefits, and toxicology of common edible flowers: a review (2000–2015). Crit Rev Food Sci Nutr.

[11] Rop O, Mlcek J, Jurikova T, Neugebauerova J, Vabkova J (2012). Edible flowers-a new promising source of mineral elements in human nutrition. Molecules.

[12] Xu YK, Liu HM, Dao XS, Xiao CF, Cai CT, Chen GH, Xu ZY (2004). Nutrients content of *Bauhinia variegata* var. candida and its value as an edible wild flower. J Yunnan Univ (Nat Sci Ed).

[13] Kumari P, Ujala, Bhargava B (2021). Phytochemicals from edible flowers: opening a new arena for healthy lifestyle. J Funct Foods.

[14] Liu XQ, Wang SY, Cui LL, Zhou HH, Liu YH, Meng LJ, Chen ST, Xi XF, Zhang Y, Kang WY (2023). Flowers: precious food and medicine resources. Food Sci Hum Wellness.

[15] Skrajda-Brdak M, Dąbrowski G, Konopka I (2020). Edible flowers, a source of valuable phytonutrients and their pro-healthy effects—a review. Trends Food Sci Technol.

[16] Amrouche TA, Yang X, Capanoglu E, Huang WS, Chen Q, Wu LP, Zhu YH, Liu YQ, Wang YX, Lu BY (2022). Contribution of edible flowers to the Mediterranean diet: phytonutrients, bioactivity evaluation and applications. Food Front.

[17] Shi YX, Zhou M, Zhang Y, Fu Y, Li JW, Yang XF (2021). Poisonous delicacy: Market-oriented surveys of the consumption of *Rhododendron* flowers in Yunnan. China. J Ethnopharmacol.

[18] Chen JL, Zhu JJ (2013). Ethnobotanical study of anthophagy culture in mountainous area of Wenzhou. Acta Agric. Jiangxi.

[19] Liu YT, Long CL. Cultural dimension in edible flowers among ethnic groups in Yunnan. Nat J (Shanghai). 2001(5): 292–297+246–310.

[20] Zheng H, Yan G, Li F (2012). Flower dietetic culture in Song Dynasty. J Beijing For Univ (Soc Sci Ed).

[21] Liu YT, Long CL (2001). Studies on edible flowers consumed by ethnic groups in Yunnan. Acta Bot Yunnanica.

[22] Liu YT, Long CL (2007). Ethnobotanical studies on the edible flowers in Lahu societies. Guihaia.

[23] Xu X, Zhang HY, Xie T, Sun QQ, Tian YL (2018). Elevational pattern of seed plant diversity in Xishuangbanna and its mechanisms. Biodiv Sci.

[24] Xu YK, Tao GD, Liu HM, Yan KL, Dao XS (2004). Wild vegetable resources and market survey in Xishuangbanna, Southwest China. Econ Bot.

[25] Pei SJ, A preliminary study of Xishuangbanna ethnobotany. Collected Papers in Tropical Botanical Research. Kunming: Yunnan People’ Press (1982).

[26] Gu W, Yang J, Yang FM, Sun QY, Wang YH, Long CL (2014). A preliminary study of traditional, wild medicinal, edible plants in Xishuangbanna, Yunnan. China Plant Divers Resour.

[27] Pei SJ. Traditional culture of flower eating on Rhododendron and Bauhinia in Yunnan, China. In: Proceeding of the international symposium on flowereating culture in Asia. Tokyo: Seibundo Shinkosha Publishing Co Ltd.; 1989. p. 18–26.

[28] Yan LC, Luo Y, Shi JP (2022). Typical wild vegetables in Xishuangbanna, Yunnan.

[29] Ulian T, Diazgranados M, Pironon S, Padulosi S, Liu U, Davies L, Howes MJR, Borrell JS, Ondo I, Pérez-Escobar OA, Sharrock S, Ryan P, Hunter D, Lee MA, Barstow C, Łuczaj Ł, Pieroni A, Cámara-Leret R, Noorani A, Mba C, Nono Womdim R, Muminjanov H, Antonelli A, Pritchard HW, Mattana E (2020). Unlocking plant resources to support food security and promote sustainable agriculture. Plants People Planet.

[30] Takahashi JA, Rezende F, Moura MAF, Dominguete LCB, Sande D (2020). Edible flowers: bioactive profile and its potential to be used in food development. Food Res Int.

[31] Zhu H, Wang H, Li BG, Zhou SX, Zhang JH (2015). Studies on the forest vegetation of Xishuangbanna. Plant Sci J.

[32] Yin CY, Yu J, Tang DY, Li HT, Li YH, Li G, Liu SF, Li XL, Mu Y (2021). Investigation on the medicinal and edible plant resources of Dai nationality in Xishuangbanna. Biotic Resour.

[33] Li Y, Li HT, Zhang ZL, Gu Z, Guo F, Zhang LX (2020). Application and analysis of medicinal plant resources of six major ethnic minorities living in Xishuangbanna. China J Chin Mater Med.

[34] Hong LY, Guo ZY, Huang KH, Wei SJ, Liu B, Meng SW, Long CL (2015). Ethnobotanical study on medicinal plants used by Maonan people in China. J Ethnobiol Ethnomed.

[35] Hu RC, Lin CR, Xu WB, Liu Y, Long C (2020). Ethnobotanical study on medicinal plants used by Mulam people in Guangxi. China J Ethnobiol Ethnomed.

[36] Tardío J, Pardo-de-Santayana M (2008). Cultural importance indices: A comparative analysis based on the useful wild plants of southern Cantabria (Northern Spain). Econ Bot.

[37] Sujarwo W, Caneva G (2016). Using quantitative indices to evaluate the cultural importance of food and nutraceutical plants: Comparative data from the island of Bali (Indonesia). J Cult Herit.

[38] Li P, Gu W, Long CL, Schmidt BM, Cheng DMK (2017). Ethnobotany of Dai people’s festival cake in Southwest China. Ethnobotany: a phytochemical perspective.

[39] Gu W, Hao XJ, Liu HX, Wang YH, Long CL (2013). Acylated iridoid glycosides and acylated rhamnopyranoses from *Gmelina arborea* flowers. Phytochem Lett.

[40] Chu YN, Lin C, Mao WH, Long CL (2022). New progress in biocultural diversity studies. Biodiv Sci.

[41] Camara-Leret R, Fortuna MA, Bascompte J (2019). Indigenous knowledge networks in the face of global change. Proc Natl Acad Sci USA.

[42] Yao RY, Heinrich M, Wei JH, Xiao PG (2021). Cross-cultural ethnobotanical assembly as a new tool for understanding medicinal and culinary values-the genus *Lycium* as a case study. Front Pharmacol.

[43] Vázquez-Martin ÁE, Aguilar-Rivera N, Filho WL, Djekic I, Smetana S, Kovaleva M (2022). Edible flora as a sustainable resource for world food. Handbook of climate change across the food supply chain.

[44] Pei SJ, Guo HJ (1989). Preliminary study on edible flowers of north-west Yunnan. Asian J Plant Sci.

[45] Xie Y, Zhang JB, Huang Y, Li Y, Yang M (2016). Preliminary studies on the Yunnan Bai people’s flower eating customs. Yunnan J Tradit Chin Med Mater Med.

[46] Zheng GY, Wen CL (2006). Study and application of floral drugs in the Compendium of Materia Medica. Asia Pac Tradit Med.

[47] Zhang XP, Bai L, Miao MS (2021). Characteristics of flower drugs in the 2020 edition of chinese pharmacopoeia. Cent. South Pharm.

[48] Ma AJX, Zou FM, Zhang RW, Zhao X (2022). The effects and underlying mechanisms of medicine and food homologous flowers on the prevention and treatment of related diseases. J Food Biochem.

[49] Yao RY, He CN, Xiao PG (2023). ‘Food and medicine continuum’ in the east and west: old tradition and current regulation. Chin Herb Med.

[50] Benvenuti S, Mazzoncini M (2020). The biodiversity of edible flowers: Discovering new tastes and new health benefits. Front Plant Sci.

[51] Cheng J (2017). On flower culture and its Chinese tradition—and the contemporary development and problems in China. Yuejiang Acad J.

[52] Zhou M, Shi YX, Bao XH, Yang SJ, Li JW, Yang XF (2019). Preliminary ethnobotanical study on edible “Tangli” flower (genus *Pyrus*) in Pu’er, Yunnan. Guihaia.

[53] Cheng GG (2021). Edible Flowers in Yunnan.

[54] Liu JB (1991). Research on the development and utilization of food colouring pigments in *Gmelina arborea*. Sci Technol Food Ind.

[55] Tai Z, Chen A, Qin B, Cai L, Xu Y (2014). Chemical constituents and antioxidant activity of the *Musa Basjoo* flower. Eur Food Res Technol.

[56] Chen XF, Yuan LB, Gu XP, Bai YJ, He XR, Gong G (2022). Research progress of chemical constituents and pharmacological activity of *Buddleja officinalis* Maxim. and quality marker predictive analysis. Chin J Inf Tradit Chin Med.

[57] Chen NH, Wei S (2017). Factors influencing consumers’ attitudes towards the consumption of edible flowers. Food Qual Prefer.

[58] Guiné RPF, Florença SG, Ferrão AC, Bizjak MČ, Vombergar B, Simoni N, Vieira V (2021). Factors affecting eating habits and knowledge of edible flowers in different countries. Open Agric.

[59] Guiné RPF, Barroca MJ, Florença SG (2018). The panorama of usage of flowers for eating purposes: results from a questionnaire survey. J Int Sci Pubs Agric Food.

[60] Rodrigues H, Cielo DP, Gomez-Corona C, Silveira AAS, Marchesan TA, Galmarini MV, Richards N (2017). Eating flowers? Exploring attitudes and consumers’ representation of edible flowers. Food Res Int.

[61] Ye Y, Leeming J (2023). Why China’s changing diet is a bellyache for public health. Nature.

[62] Lucas E, Guo M, Guillen-Gosalbez G (2023). Low-carbon diets can reduce global ecological and health costs. Nat Food.

[63] Chen H, Shen J, Xuan JQ, Zhu AN, Ji JS, Liu XR, Cao YY, Zong G, Zeng Y, Wang XX, Yuan CZ (2022). Plant-based dietary patterns in relation to mortality among older adults in China. Nat Aging.

[64] Li M, Jia N, Lenzen M, Malik A, Wei L, Jin Y, Raubenheimer D (2022). Global food-miles account for nearly 20% of total food-systems emissions. Nat Food.

